# Robust Vaginal Colonization of Macaques with a Novel Vaginally Disintegrating Tablet Containing a Live Biotherapeutic Product to Prevent HIV Infection in Women

**DOI:** 10.1371/journal.pone.0122730

**Published:** 2015-04-14

**Authors:** Laurel A. Lagenaur, Iwona Swedek, Peter P. Lee, Thomas P. Parks

**Affiliations:** 1 Osel, Inc., Mountain View, California, United States of America; 2 Vaccine Branch, National Cancer Institute, National Institutes of Health, Bethesda, Maryland, United States of America; University of California, San Francisco, UNITED STATES

## Abstract

MucoCept is a biotherapeutic for prevention of HIV-1 infection in women and contains a human, vaginal *Lactobacillus jensenii* that has been genetically enhanced to express the HIV-1 entry inhibitor, modified cyanovirin-N (mCV-N). The objective of this study was to develop a solid vaginal dosage form that supports sustained vaginal colonization of the MucoCept *Lactobacillus* at levels previously shown, with freshly prepared cultures, to protect macaques from SHIV infection and to test this formulation in a macaque vaginal colonization model. Vaginally disintegrating tablets were prepared by lyophilizing the formulated bacteria in tablet-shaped molds, then packaging in foil pouches with desiccant. Disintegration time, potency and stability of the tablets were assessed. For colonization, non-synchronized macaques were dosed vaginally with either one tablet or five tablets delivered over five days. Vaginal samples were obtained at three, 14, and 21 days post-dosing and cultured to determine *Lactobacillus* colonization levels. To confirm identity of the MucoCept *Lactobacillus* strain, genomic DNA was extracted from samples on days 14 and 21 and a strain-specific PCR was performed. Supernatants from bacteria were tested for the presence of the mCV-N protein by Western blot. The tablets were easy to handle, disintegrated within two minutes, potent (5.7x10^11^ CFU/g), and stable at 4°C and 25°C. Vaginal administration of the tablets to macaques resulted in colonization of the MucoCept *Lactobacillus* in 66% of macaques at 14 days post-dosing and 83% after 21 days. There was no significant difference in colonization levels for the one or five tablet dosing regimens (p=0.88 Day 14, p=0.99 Day 21). Strain-specific PCR confirmed the presence of the bacteria even in culture-negative macaques. Finally, the presence of mCV-N protein was confirmed by Western blot analysis using a specific anti-mCV-N antibody.

## Introduction

MucoCept is a recombinant live biotherapeutic product (rLBP) containing the human vaginal *Lactobacillus* strain, *L*. *jensenii* 1153–1666, which was genetically engineered to secrete a modified form of the potent HIV-1 entry inhibitor protein, cyanovirin-N (mCV-N) [1]. MucoCept is being developed as a novel live microbicide/biotherapeutic to prevent HIV infection in women.

The human vaginal microbiota plays a key role in preventing a number of sexually transmitted infections (STIs), including human immunodeficiency virus type 1 (HIV-1) [2–6]. Surfaces of the human vagina and cervix are colonized chiefly by *Lactobacillus*, which protect the mucosa from urogenital pathogens and contribute to women’s reproductive tract. The most common species of vaginal lactobacilli in women worldwide are *L*. *crispatus*, *L*. *iners*, *L*. *jensenii*, and *L*. *gasseri* [7, 8]. Hydrogen peroxide and lactic acid-producing species, predominantly *L*. *crispatus* and *L*. *jensenii*, have been inversely associated with bacterial vaginosis, STIs, and HIV-1 acquisition [2–5, 9].

A rhesus macaque model was developed for testing the safety and efficacy of this uniquely human vaginal strain [10]. Rhesus macaques harbor distinct strains of lactobacilli that are easily distinguishable by colony morphology and biochemically from human *L*. *jensenii*, most often *L*. *johnsonii* or *L*. *reuteri* [10, 11]. We have shown that *L*. *jensenii* 1153–1666 is capable of colonizing the vaginal mucosa of rhesus macaques and producing mCV-N *in situ* for greater than six weeks after a five day dosing period [12]. Persistent colonization and mCV-N production by the recombinant strain does not activate inflammatory cytokines IL-1β, IL-6, TNFα, GM-CSF, IFN-γ, IL-8, IL12/23(p-40), IL-17, and IL-18, measured prior to and during repeated challenges with a chimeric simian-human immunodeficiency virus (SHIV) [13]. We previously showed that macaques colonized with the highest levels of *L*. *jensenii* 1153–1666, at least 10^6^ colony forming units (CFU), had lower vaginal pH by an estimated 0.4 pH units from those with no *Lactobacillus* colonization (p = 0.0058 by repeated measures analysis of variance) [13].

Macaques dosed with *L*. *jensenii* 1153–1666 exhibited a 63% reduction in the rate of acquisition of the SHIV_SF162P3_ virus after repeated vaginal challenges and a six-fold reduction in peak viral load in animals with breakthrough infection [12]. In all previous studies, the rLBP was delivered as a freshly prepared live bacterial culture mixed with 3% hydroxyethylcellulose.

The aim of this study was to develop a potent and stable solid vaginal dosage form for MucoCept that can support vaginal colonization of *L*. *jensenii* 1153–1666 as well as an inoculum of freshly cultured cells. Only a small amount of fluid is present on vaginal mucosal surfaces. Conventional tablets or capsules are designed primarily for oral administration and dissolve relatively slowly in the vagina and therefore are not optimal for vaginal delivery of LBPs. An LBP vaginal dosage form should disintegrate quickly on the mucosa so bacteria can rehydrate and begin growing to establish themselves within the vaginal microbiota. Orally disintegrating tablets (ODTs) have been developed that dissolve rapidly on the buccal mucosa or tongue in the presence of saliva only and do not require swallowing, which is difficult for many individuals [14]. The ODT concept also applies to vaginal drug delivery where little fluid is available for tablet disintegration. However, analogous vaginally disintegrating tablet (VDT) for delivering LBPs are not currently available. Effervescent *Lactobacillus* containing tablets with both fast and sustained release properties have been described [15] and tested for treatment of bacterial vaginosis [16, 17]. In this proof of concept study we produced prototype VDTs that were potent, easy to handle, and maintained potency when stored at 4°C or 25°C. Importantly, vaginal administration of the MucoCept VDTs resulted in robust and sustained colonization with *L*. *jensenii* 1153–1666 at levels previously shown to protect macaques from SHIV infection. These results suggest that MucoCept VDTs may be an effective and convenient product that women could self-administer in a discreet and coital-independent manner to help prevent HIV-1 infection.

## Materials and Methods

### Tablet preparation

*L*. *jensenii* 1153–1666 was grown in 1 L of modified* Mann Rogosa Sharp (MRS) medium to early stationary phase in a BioFlo 110 Bioreactor (New Brunswick/ Eppendorf Inc., Enfield CT) at 37°C and pH 6.0. *The standard Mann, Rogosa, and Sharpe medium used to propagate *Lactobacillus* was modified to replace proteose peptone and beef extract with alternative components that do not contain animal byproducts.

The cells were harvested by centrifugation (1500 x g for 10 min), washed with phosphate buffered saline, and resuspended in a proprietary preservation matrix containing skim milk, trehalose, xylitol, and sodium ascorbate. The mixture was dispensed into tablet-shaped molds and lyophilized in a Virtis Advantage Lyophilizer (SP Scientific, Warminster, PA). The resulting 10x15 mm tablets were suitable for vaginal insertion in macaques to test colonization ability. The tablets were stored in heat-sealed Mylar foil pouches with a desiccant (MiniPax, Multisorb Technologies) at 4°C, 25°C, or 37°C for up to one year. Two batches of prototype VDTs were prepared that were nearly identical in appearance, weight, and potency.

### Tablet characteristics

*Weight*: Eight randomly selected tablets were weighed on an Ohaus Analytical Plus precision balance. *Potency*: Tablets were weighed and dissolved in 10 ml of MRS. Serial dilutions were performed and bacteria plated in triplicate onto MRS agar plates. The plates were incubated at 37°C for 48 hr. under anaerobic conditions (GasPak EZ Anaerobe Pouch System). Colonies were counted manually and the final number was multiplied by the appropriate dilution factor to yield the potency. Potency was expressed as the total CFU normalized to tablet weight (CFU/g). *In vitro Disintegration Time*: In the absence of a standardized dissolution test for tablets that rapidly disintegrate on mucosal surfaces, we used the simulated wetting test that has been shown to closely mimic disintegration of orally disintegrating tablets in the mouth [18]. Five layers of natural unbleached paper towel (Seventh Generation brand) were placed in a 10-cm diameter Petri dish and 10 ml of vaginal fluid simulant (NaCl, 3.51g/L; KOH, 1.40 g/L; Ca(OH)_2_, 0.222 g/L; bovine serum albumin, 0.018 g/L; lactic acid, 2.00 g/L; acetic acid, 1.00 g/L; glycerol, 0.16 g/L; urea, 0.4 g/L; and glucose, 5.0 g/L) [19]. Tablets were placed on the surface of the tissue paper in the Petri dish at 25°C and the time required for vaginal fluid simulant solution to completely wet the tablet surface was recorded using a stopwatch. These measurements were carried out with eight tablets.

### Study Design

The rhesus macaque is a suitable model for testing *Lactobacillus*-based therapeutics because the vaginal anatomy and physiology is similar to humans and their vaginal microbiota often harbors endogenous strains of lactobacilli [10, 11]. The purpose of this pilot study was to test the hypothesis that MucoCept VDTs can vaginally colonize macaques at similar levels to those seen with freshly prepared bacteria. Two dosing regimens were to be compared, a single tablet vs. five tablets administered over five days. This pilot study was not powered to detect differences between the one-tablet arm and the five-tablet arm. Our hypothesis was that administration of five-tablets would result in better colonization than one-tablet. The five-tablet group was assigned 10 macaques, while the one-tablet group was assigned eight macaques. Menses were monitored one week before and throughout the study and used to determine the phase of the menstrual cycle.

### Macaque vaginal colonization

The eighteen sexually mature (ages 3–13) female Chinese origin rhesus macaques (Macaca mulatta), used in this study were housed at BIOQUAL, Inc., in accordance with the recommendations of the Association for Assessment and Accreditation of Laboratory Animal Care International Standards and with the recommendations in the Guide for the Care and Use of Laboratory Animals of the United States—National Institutes of Health. The Institutional Animal Use and Care Committee of BIOQUAL approved these experiments IACUC # 11-3443-86.

BIOQUAL's animal facilities meet or exceed all AAALAC, USDA and OLAW standards for animal housing, and the surrounding environment. Each room can hold approximately 20–34 NHPs in open 6.0 sq.ft. mobile home over/under modules. The cages have shuttle doors on both sides, which allow the cages to "lock" together and thus provide pair housing when allowed by the protocol, and also allows for easy transfer of each animal at the bi-weekly cage change time. In addition, 9.0 sq.ft. mobile activity/observation modules, either independently or connected to a 9.0 standard mobile home over/under modules are available and have been used for SIV animals with special medical or behavioral needs.

BIOQUAL normal weekday working hours for the primate housing facilities are from 7–4 pm. Technician performs animal health checks and provides medications when needed. The animal caretakers feed and water the animals.

Animals requiring therapy or observations at six, eight, or twelve-hour intervals have a technician scheduled to return to the vivarium as required to ensure proper treatment. All animals are observed closely for development of clinical signs. Observations are done twice a day, seven days a week by the assigned nonhuman primate technicians as per SOP 112. Any abnormality is noted on a health status check sheet and brought to the attention of the veterinarians (SOP 101). If an animal requires medication, a treatment form is filled out by the veterinarian designating what medication to give, how often and the route of administration. The technician giving the medication is required to initial the treatment form. This information is also written in the animal’s record. The COR will be notified of any untoward findings. No medication will be administered without approval of the COR. BIOQUAL provides the following coverage: Monday through Friday, from 6:00 a.m. to 4:00 p.m. the animals are observed hourly; Monday through Friday, from 4:00 p.m. to 6:00 a.m. room temperatures are recorded and the animals are observed if required due to medical concerns; in general we do not turn on the lights and disturb the animals during the dark cycle (6:00 PM—6:00 AM). Saturdays, Sundays and holidays from 7:30 a.m. to 11:30 a.m. the animals are observed hourly, from 12:00 p.m. (noon) to 7:00 a.m. room temperatures and humidity are recorded and the animals are observed as required. Experienced technical and professional staff assures consistent subjective assessment of the animals during a study to complement the objective clinical data. Experience is also critical for effective response to the clinical emergencies of infected animals thus preventing the loss of important data and precluding discomfort of the animal. Any animal requiring more frequent observation or treatment has an experienced animal technician and/or veterinarian come in as required to provide care, 24/7.

The physical examination includes body weight, body temperature, pulse and respiratory rates. In addition, abdominal palpation with notation of any enlargement of the liver, the spleen and other abdominal change are also noted. Any changes in external appearance are recorded. Peripheral lymph nodes are also examined. Daily records of food consumption by amount, general activity and the condition of bowel movements are maintained for animals with clinical signs. Diagnostic clinical pathology blood samples are drawn as the animals' clinical health requires and all USDA-covered species (NHPs and ferrets) are given quarterly physical examinations. Complete blood counts (CBC), serum chemistries, and blood clotting profiles are performed by commercial laboratories (IDEXX) or by trained personnel (clotting profiles). BIOQUAL has a licensed biohazardous materials courier service to rapidly deliver samples from the vivarium to BIOQUAL laboratories, commercial laboratories such as IDEXX laboratories or the NIH.

Veterinarian and technician on call schedules are posted prominently at all BIOQUAL facilities. There is a minimum of one trained and experienced technician for each of the BIOQUAL facilities on call 24 hours a day, seven days per week, for any animal emergency or necessary medical treatments. The veterinarians similarly provide emergency coverage 24 hours per day, seven days a week, on a rotating basis. The on call schedules include home telephone numbers, cell phone numbers, and pager numbers so that the veterinarian and technicians on call at that time can be contacted. No technicians or veterinarians are added to the on call schedule until they are thoroughly familiar with all work performed at BIOQUAL facilities, are comfortable with, and have shown they can perform, any procedure they may need to do after hours or on an emergency basis.

The animals were provided with a commercial primate diet and fresh fruit twice daily, with water freely available at all times. All NHPs housed at BIOQUAL, Inc. receive bi-monthly behavioral assessments from one of two fulltime behaviorists. All NHPs receive at least three commercially available pet toys in their cages at all times to manipulate as part of their enriched environment. Choosing from a wide array of toys, each animal is provided one hard toy (i.e. hard plastic), one soft toy (i.e. soft rubber), and a third toy (i.e. hard plastic or soft rubber) chosen by the caretaker assigned to each animal. NHPs are given destructible enrichment as a way for them to alter their environment, release aggression/tension, and forage through. The destructible enrichment provided includes items such as cardboard, shredded paper, and phonebooks. Soft toys, such as fleece blankets and soft pet toys, are available for comfort to all NHPs. Many foraging opportunities are made available to all NHPs. Treats used for foraging and in feeders include, but are not limited to: Bunny Blocks, Nutra Blocks, Crumble Disks, PRIMA Treats, Fruity Gems, Fleece Foraging Crumbles, wax moth larvae (all available through BIOSERV), raisins, popcorn, nuts, birdseed, oatmeal, hardboiled eggs, species-specific food items, etc. All animals observed to exhibit any abnormal behavior(s) deemed detrimental to the physical and/or mental health of the individual are prescribed specific enrichment by the behavioral staff in order to decrease/eliminate/redirect such behaviors.

The experiments were Level C: No more than momentary or slight pain or distress. When immobilization was needed, the animals were sedated by intramuscularly injection with 10 mg/kg of ketamine HCl and 1 mg/kg acepromazine to alleviate stress. Details of animal welfare and steps taken to ameliorate suffering were in accordance with the recommendations of the Weatherall report, “The use of non-human primates in research”.

Macaques were not synchronized with hormones and no antibiotics were used prior to or during the experiment. Female macaques were trained to present their external genitalia to the veterinary staff and menses was monitored by insertion of a cotton tip swab into the vagina and quantifying menstrual blood as 1+ trace blood, 2+ medium, 3+ moderate. Menstruation was observed prior to and throughout the study, but the animals were not synchronized. Macaques were dosed vaginally with either one MucoCept tablet (Group 1 received one tablet, n = 8) or one tablet daily for five consecutive days (Group 2 received five tablet, n = 10). The vaginal microbiota of each animal was sampled prior to tablet administration, and in both groups at 14 and 21 days post-dosing. Group 1 animals were also sampled at day three post-dosing. To sample the microbiota, two swabs were inserted to the vaginal fornix, and then placed into a Port-A-Cul anaerobic collection and transport tube (Becton Dickinson, Cockeysville, MD, USA). Animals in follicular phase (F) or luteal phase (L) at the time of tablet administration are noted, when accurate determination could be made. We were not able to accurately determine the stage of cycle for all macaques.

### Microbiology culture conditions

For microbiological analyses, two vaginal swabs were collected from each macaque and transported to the laboratory for processing within 4 hrs. One swab was placed in 1 ml of phosphate buffered saline, pH7.4 (Quality Biological, Inc., Gaithersburg, MD) and mixed with a vortex mixer. Serial dilutions were carried out, followed by plating on Rogosa SL agar (Difco, Sparks, MD). The second swab was used for direct extraction of genomic DNA (see below). Plates were incubated 48 hr. at 37°C under 5% CO_2_ (BD GasPak EZ CO_2_ Pouch System, Becton Dickinson) and *L*. *jensenii* colonies were counted. *L*. *jensenii* colonies can be readily discriminated from endogenous *Lactobacillus* species found in the macaque.

### *L*. *jensenii* 1153–1666 identification

Gram stain was first performed on characteristic *L*. *jensenii* colonies. Genomic DNA was extracted from representative *Lactobacillus* colonies from each macaque to confirm bacterial identification using a Mo Bio PowerSoil Genomic DNA isolation Kit (Mo Bio Laboratories, Inc., Carlsbad, CA). In addition, genomic DNA was extracted directly from the second vaginal swab at days 14 and 21. Bacterial identification was confirmed using polymerase chain reaction (PCR) of a specific 247 base pair genomic fragment containing portions of the *pox*1 gene chromosomal integration site and the mCV-N expression cassette. These sequences are only present in the recombinant *L*. *jensenii* 1153–1666 strain. Construction of the *L*. *jensenii* 1153–1666 strain is described in Liu *et al* [1]. PCR primers CVN-Forward (5'-TCAGAATTGGCTGCTGAATG-3') and CVN-reverse (5'-TGGCTCGCTACAATATGCAC-3') were added to PCR Supermix High Fidelity (Life Technologies, Invitrogen, Grand Island, NY) and 1–2 μl of extracted genomic DNA to amplify the 247 base pair fragment. Direct PCR from swabs was not performed at day three.

### Cyanovirin-N detection

Strains of *L*. *jensenii* 1153–1666, from macaque vaginal samples, were grown in 1 ml of MRS broth overnight at 37°C under 5% CO_2_. Bacterial supernatants were separated from the bacterial cells by centrifugation and 30 μl of each supernatant sample was loaded on a 12% Bis-Tris gel (Life Technologies) and transferred to a PVDF membrane using the iBLOT Dry Transfer System (Life Technologies). Protein was detected with anti-CV-N rabbit primary antibody, as previously described [1], using an anti-rabbit Western Breeze Chromogenic Immunodetection System (Life Technologies). Detection of mCV-N in cervicovaginal lavage samples from macaques colonized with *L*. *jensenii* 1153–1666 has previously been described[12].

### Statistical analysis

Statistical analysis was performed with Statistical Analysis System (SAS) software using a one-sided Wilcoxon Rank Sum test.

## Results

### Tablet characteristics

**Fig 1:**
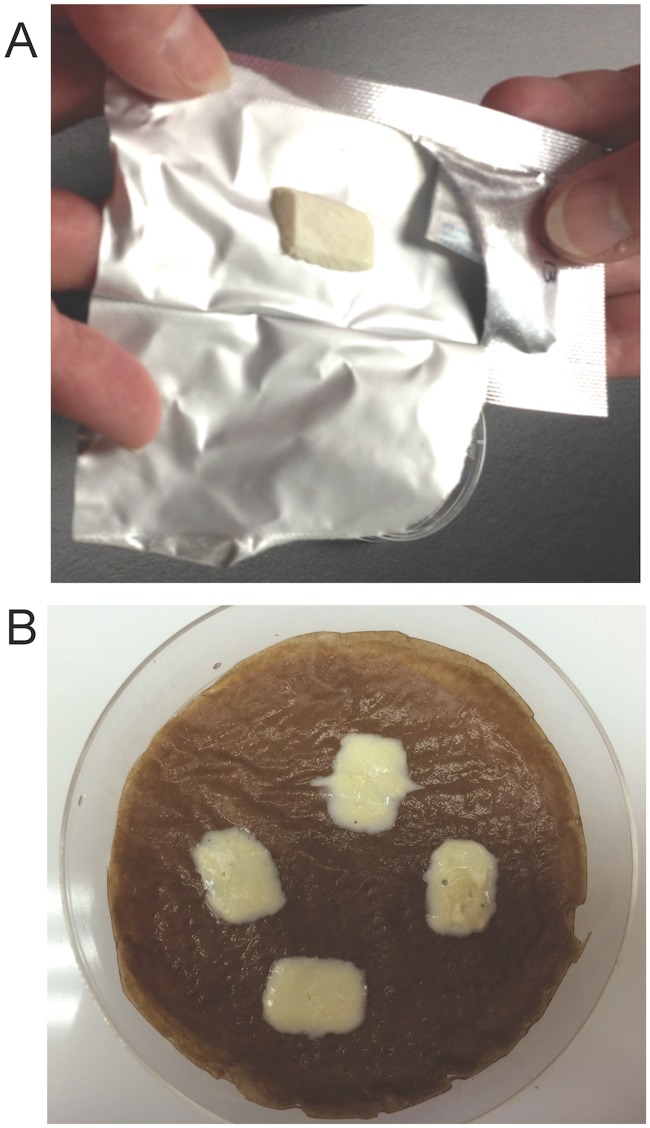
MucoCept prototype VDT before and after disintegration. A) Mylar foil pouch opened to reveal tablet and desiccant. B) Tablet disintegration is shown at three minutes. The tablet is placed on a paper towel wetted with vaginal fluid simulant, to simulate the mucosal surface. The time required for the solution to completely wet the tablet surface was measured as the disintegration time. Tablets disintegrated within two minutes.

*L*. *jensenii* 1153–1666 cells were formulated in a proprietary preservation matrix and lyophilized in tablet-shaped molds to yield prototype MucoCept tablets that were suitable for testing in the macaque vaginal colonization model (Fig 1A). The tablets could be stored and handled without breaking, disintegrated in less than 2 minutes *in vitro* (Fig 1B), and weighed 113.4±2.1 milligrams (mg) (mean ± standard deviation, n = 8).

### Tablet potency and stability

**Fig 2:**
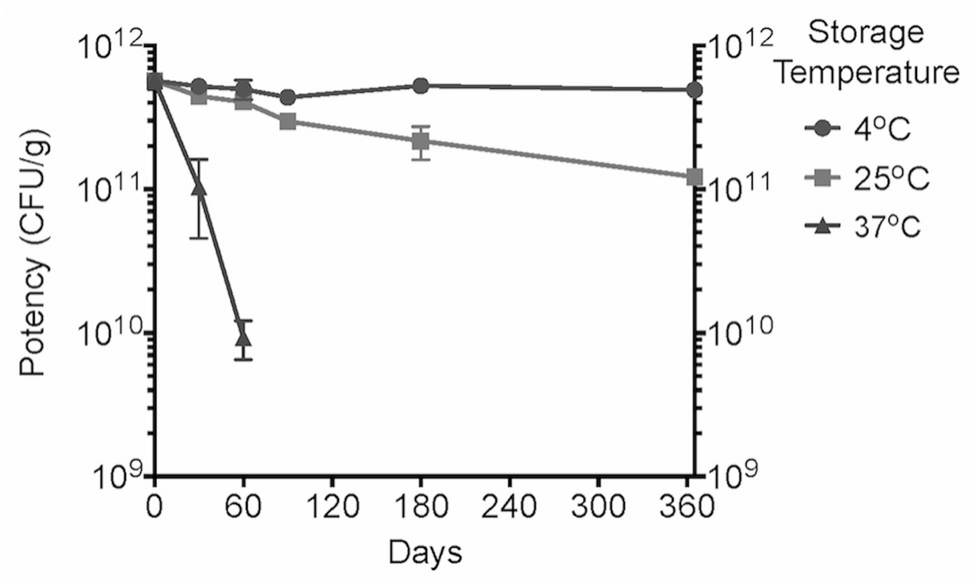
Stability of MucoCept VDTs. Two batches of tablets were prepared and placed in storage at 4°C, 25°C, or 37°C. Potency was measured as CFU/g at time zero, one, two, three, six, and 12 months to assess stability. The mean initial potency of the tablets was 5.7x10^11^ CFU/g. Error bars represent the standard error of the mean (SEM). The potency of tablets stored at 4°C and 25°C for one year was 4.9x10^11^ CFU/g and 1.2x10^11^ CFU/g, respectively. Tablets stored at 37°C lost 1.8 log_10_ CFU/g of their initial potency after two months (to 9.4x10^9^ CFU/g). Stability at 37°C was discontinued after two months.

Two batches of MucoCept VDTs were prepared for evaluation of potency and stability. The two batches of tablets were quite similar in appearance, weight, potency, and stability. The mean initial potency of the tablets was 5.7x10^11^ CFU/g and a potency of ≥1.0x10^11^ CFU/g could be maintained for at least one year when stored at 4°C or 25°C (Fig 2). The activity of tablets stored at 37°C could be maintained at ≥1.0x10^11^ CFU/g for only one month. It should be noted that the preservation matrix used to prepare the prototype tablets was not optimized for maximal stability since a primary goal of the study was to establish feasibility of the MucoCept VDT dosage form.

### Colonization of *L*. *jensenii* 1153–1666 in macaques

The rhesus macaque model is commonly used for microbicide and vaccine studies, but has not been used for testing live biotherapeutic products. We previously characterized the model to determine whether it was suitable for colonization studies with human vaginal strains of *Lactobacillus* and we found that rhesus macaques of Chinese and Indian origin often harbor endogenous vaginal lactobacilli at low levels and can support the growth of human vaginal strains [10]. In this study we used the model to determine whether the MucoCept vaginal tablets were capable of achieving the same level of colonization (≥10^7^ CFU/swab) of MucoCept bacteria previously observed using fresh cultures mixed with 3% hydroxyethylcellulose. Our hypothesis was that colonization would be superior in the five-tablet group compared to the single-tablet group.

**Fig 3:**
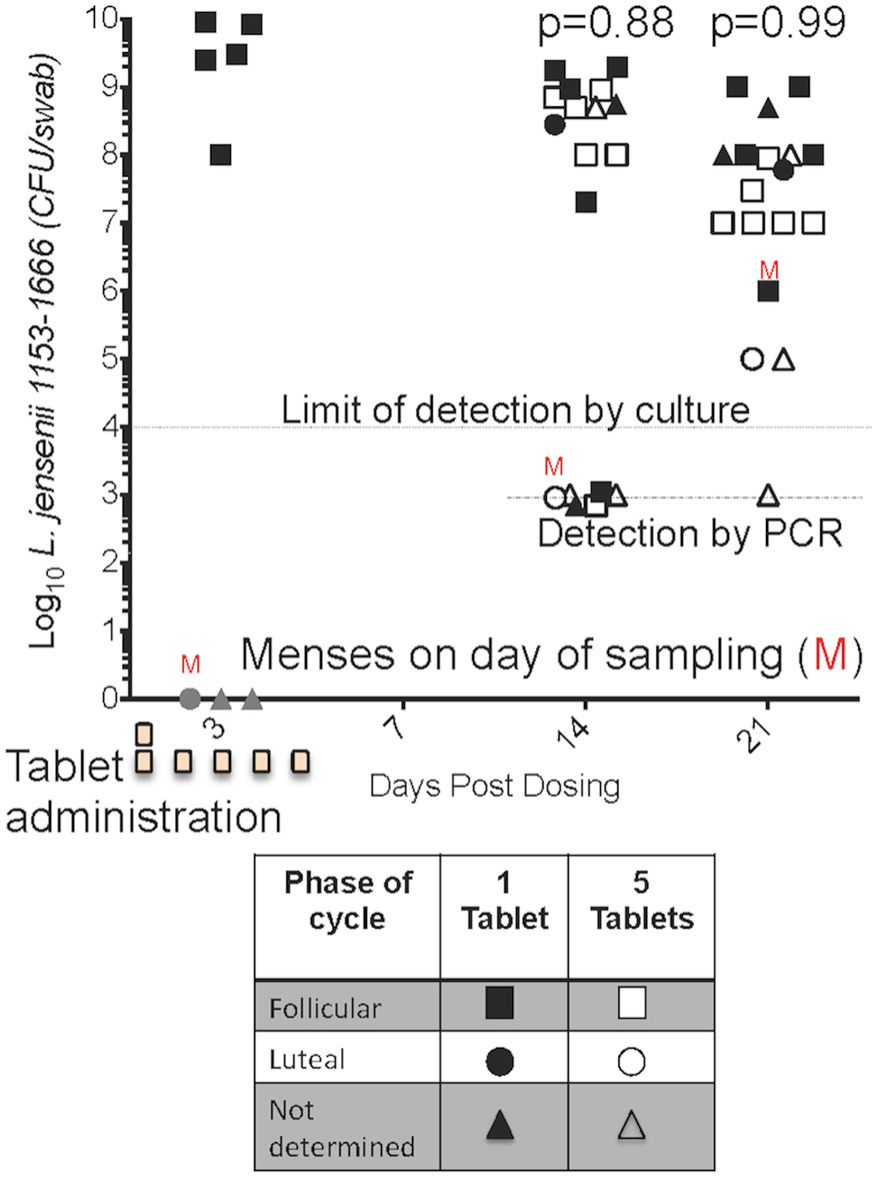
Vaginal colonization of macaques following administration of MucoCept VDTs. Macaques were vaginally administered one (closed symbols) or five tablets (open symbols) and colonization of *L*. *jensenii* 1153–1666 determined at days 14 and 21 post-dosing. In addition, macaques dosed with one tablet were swabbed after three days to determine the initial delivery of viable bacteria to the vagina. Colonization was expressed as CFU/swab following growth of vaginal bacterial dilutions on Rogosa agar and colony counting. Menses (M) is noted in three macaques on the day of sampling. Animals in follicular phase or luteal phase at the time of tablet administration are noted when accurate determination was possible. No significant difference was found between one tablet and five tablet administration at day 14 p = 0.88 or day 21 p = 0.99 based on a one sided Wilcoxon Rank Sum test.

Following tablet administration, vaginal swabs were collected for MucoCept bacterial colony counts at day three, 14 and 21 and for MucoCept strain-specific PCR at day 14 and 21(Fig 3). High levels of *L*. *jensenii* 1153–1666 (4.6±3.9x10^9^ CFU/swab) were observed three days post-dosing in five of eight macaques that received one tablet, indicating that the tablets successfully delivered viable bacteria to the lower reproductive tract. Three macaques had undetectable *L*. *jensenii* 1153–1666 levels by culture at day three; menses was noted in one macaque. We waited until days 14 and 21 post-dosing to allow the bacteria sufficient time to become established in the vaginal microbiota. The drop in colony counts from day three to day 21 reflects the high level of viable bacteria initially delivered to the vagina followed by their establishment within the vaginal ecosystem at sustainable levels. At day 14 post-dosing 67% (n = 12) of animals were highly colonized (≥10^6^ CFU/swab) with a mean of 4.6±6.1x10^8^ CFU/swab (mean±standard deviation). At day 21, 83% (n = 15) of macaques were highly colonized with a mean of 1.7±3.2x10^8^ CFU/swab (mean±standard deviation). Colonization levels of *L*. *jensenii* 1153–1666 were considerably lower (~1x10^5^ CFU/swab) in two macaques, and detectable only by PCR in one macaque (limit of detection was 1000 CFU/swab), at day 21 post-dosing. The levels of colonizing *L*. *jensenii* 1153–1666 with the single tablet dosing in this study were comparable to the five tablet-dosing regimen. No statistical difference was found between the groups at days 14 and 21 using one-sided Wilcoxon Rank Sum test (p = 0.88 Day 14, p = 0.99, Day 21). The colonization levels were consistent with the levels of endogenous lactobacilli found in rhesus macaques (average 10^5^–10^7^ CFU/swab) and achieved with freshly prepared and vaginally inoculated bacteria mixed in hydroxyethylcellulose in our previous studies. Follicular or luteal phase, at the time of tablet administration, is noted when they could accurately be determined. A majority of the well-colonized macaques were in the follicular phase at the time of tablet administration. Two macaques in luteal phase also appeared to be well colonized. Our original hypothesis the five tablet administration would result in more colonization than the one tablet administration was not supported.

### Identification of *L*. *jensenii* 1153–1666 in macaques

**Fig 4:**
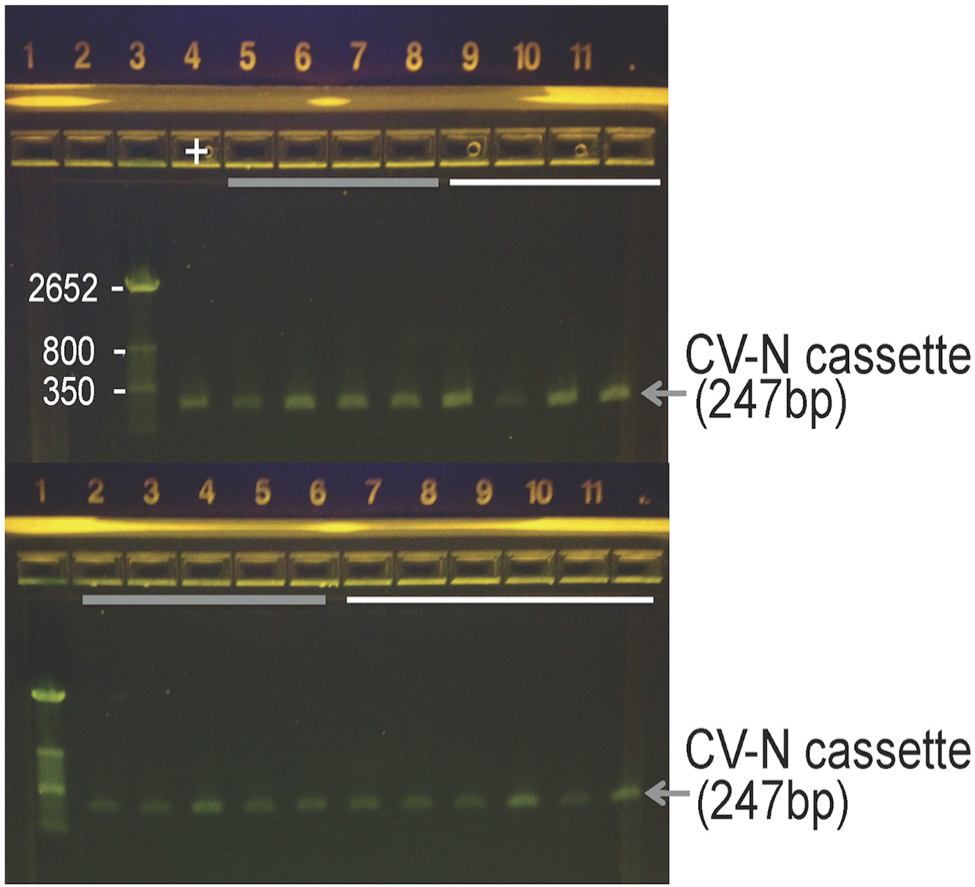
Identification of *L*. *jensenii* 1153–1666 confirmed by strain-specific PCR. Genomic DNA was extracted from vaginal swabs and PCR performed using *L*. *jensenii* 1153-1666-cyanovirin cassette specific primers. All the macaques were PCR-positive at 21 days post-inoculation as indicated by the presence of 247 base pair PCR fragment that uniquely identifies the recombinant strain. The solid grey lines indicate macaques that received one tablet; solid white lines represent macaques that received five tablets.

**Fig 5:**
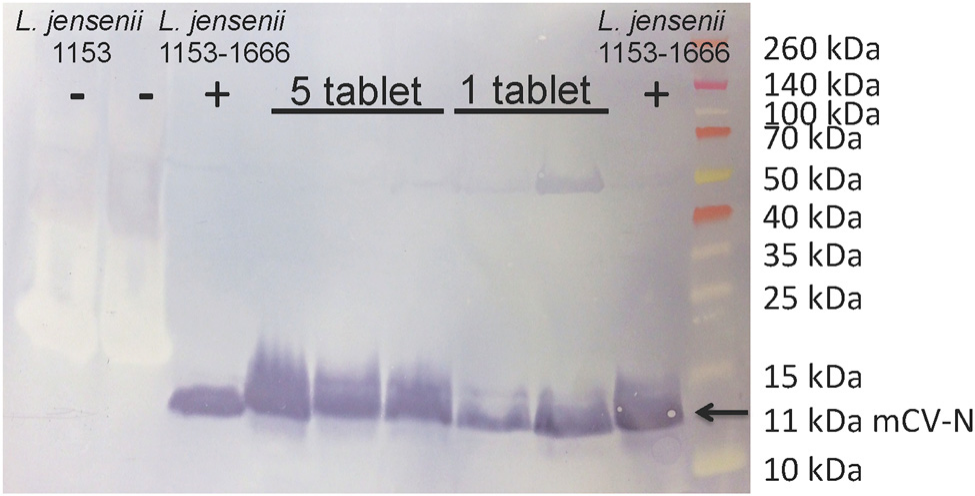
Western blot to confirm mCV-N protein expression. Proteins in stationary phase conditioned media from *L*. *jensenii* 1153–1666 isolated from macaques that received one-tablet or five-tablets were separated on a 12% Bis-Tris gel. A representative gel is shown from the day 14-post dosing time point. The parental strain, *L*. *jensenii* 1153, is shown as the negative control, and *L*. *jensenii* 1153–1666 as the positive control. Three macaques from the five-tablet group and two macaques from the one-tablet group are shown, confirming that colonies of *L*. *jensenii* 1153–1666 recovered from the macaques continued to produce mCV-N protein.

Macaques do not naturally harbor *L*. *jensenii*, which is unique to the human microbiota; *L*. *johnsonii* and *L*. *reuteri* are instead part of the vaginal microbiota of the macaque [10, 11]. *L*. *jensenii* 1153–1666 contains the *mCV-N* gene within the bacterial chromosome, which permits the strain to be unambiguously identified by PCR [1]. To confirm the identify *L*. *jensenii* 1153–1666, we first isolated bacterial colonies, performed Gram-stain to test for Gram-positive rods, and extracted the genomic DNA. PCR was performed with two primers that are specific to the mCV-N expression cassette and flanking genomic integration site. PCR reactions were run on a 1.2% agarose gel and the presence of a specific 247 base pair band positively identified the MucoCept strain. These results were confirmed by PCR on genomic DNA extracted directly from vaginal swabs collected at days 14 and 21. PCR fragments containing the mCV-N cassette are shown in Fig 4, confirming the presence of *L*. *jensenii* 1153–1666 in all the macaques at day 21 post-dosing. In addition, the ability of *L*. *jensenii* 1153–1666 to produce mCV-N, an important functional phenotype, was confirmed by Western blot (Fig 5). A representative blot from Day 14 is shown.

## Discussion

MucoCept is a novel genetically engineered LBP that combines recent advances in our understanding of the vaginal microbiome and its relationship to vaginal health, with the active prevention of HIV infection at a mucosal site of virus entry using a vaginal strain that constitutively secretes a potent HIV entry inhibitor, mCV-N [20]. Modified CV-N was selected as an HIV inhibitor because it has broad activity against diverse HIV-1 subtypes, including subtype C that predominates in South Africa [20–23]. Modified CV-N is not used in primary HIV therapy. CV-N resistant strains of HIV-1 have been experimentally developed in the laboratory and shown to have an increased sensitivity to antibody neutralization due to loss of the glycan shields on gp120 [24].

The MucoCept LBP uses a commonly found vaginal species of *L*. *jensenii*. Although variations of the microbiota exist within individuals over time and in different geographical settings, *L*. *jensenii* is found in 17%-41% of women across many regions of the world [7, 25–33]. Study of additional factors that influence colonization in women, such as bacterial vaginosis [34], estrogen and glycogen levels [35], especially in areas where this product’s use in intended, will be necessary. The development of MucoCept as a formulated tablet has been facilitated by the experience gained from LACTIN-V, a *Lactobacillus crispatus*-based LBP in Phase 2 clinical development by Osel, Inc. [36, 37]. The ability to modulate the microbiome and enhance its beneficial properties represents a new wave of potential products for improving human health and preventing disease.

Even with proven efficacy [38, 39] and high acceptability of pre-exposure prophylaxis (PrEP), adherence to a daily product protocol has proven challenging and is a major factor for achieving product efficacy in preventing HIV infection [40–43]. Novel dosage forms such as once-monthly vaginal rings to deliver drug are being actively tested [44] and it is hoped that they will lead to higher adherence than was found in the FEM-PrEP, VOICE [45, 46] and FACTS-001 studies. MucoCept delivered as a vaginally disintegrating tablet, containing a colonizing, *Lactobacillus* could be self-administered in a discreet, coitally independent manner, making it convenient and easy to use.

Here we demonstrate the feasibility of delivering a novel solid dosage form of a recombinant live biotherapeutic, a vaginally disintegrating tablet, and show proof of concept that the VDTs could deliver equivalent levels of *L*. *jensenii* 1153–1666 to the macaque vagina as we did with freshly prepared bacteria. The prototype tablets used in this study exhibited sufficient stability for testing *in vivo*, and could withstand one month storage at 37°C. However, for use in the developing world, where cold chain storage may not be readily available, the formulation will need to be optimized to improve the product’s stability at elevated temperatures.

The tablets were easy to handle, dissolved rapidly *in vitro*, exhibited high potency, and resulted in high level vaginal colonization of macaques following vaginal administration of as few as one tablet. Based on these results, we expect MucoCept VDT to be at least as effective in preventing SHIV infection in the macaque model as observed previously [12]. Vaginal colonization of *L*. *jensenii* 1153–1666 in macaques is a useful surrogate marker for protection from SHIV infection with ≥ 10^6^ CFU/swab being correlated with protection. The amount of mCV-N produced *in vivo* is proportional to the colonization levels [1] and at ≥ 10^6^ CFU/swab mCV-N can be readily detected in macaque cervicovaginal lavage samples [12]. We previously reported that endogenous *Lactobacillus* in macaques varied from 10^3^ CFU/swab at menses, when levels were lowest, to 10^7^ CFU/swab [10].

Macaques in this study were not synchronized with hormones and were sampled on specific days, thus several animals were menstruating on the day of sampling. We previously showed that the levels of lactobacilli in the macaque vagina can drop as much as four-logs during menses [10]. Two of the samples collected during menses were below the level of detection by culture. A small number of macaques did not exhibit high-level colonization for reasons that remain unclear. Colonization may have occurred more slowly in these animals most probably due to physiological differences between the macaques and humans. It has been reported that rhesus macaques have significantly lower genital tract glycogen, which is a key carbon source for vaginal lactobacilli [35] and these macaques may have been deficient in vaginal glycogen.

Although no significant difference, in colonization levels, was seen for the one or five tablet dosing regimens, the small numbers of animals used in this exploratory study may not have been sufficient to detect a difference. However, we can say that we do not have evidence to support our original hypothesis that the five-tablet arm would be colonized at higher levels based on the data presented in Fig 3. Menstruation on different days in the study groups may also have contributed to variations.

Prevention strategies, such as microbicides and topically applied antiretroviral drugs also known as pre-exposure prophylaxis (PrEP), are designed to interdict HIV infection at the mucosal surface and represent approaches to reduce the risk of HIV infection. Orally delivered Truvada (a fixed dose combination of emtricitabine and tenofovir disoproxil fumarate) has been approved by the Food and Drug Administration for pre-exposure prophylaxis against HIV infection. Although drug resistance was rare in the IPrEx study [47], concerns remain over the use of front-line antiviral drugs as microbicides because of the potential for development of viral resistance. In addition, there are ethical concerns regarding the circulation of antiretroviral drugs (ARVs) to healthy individuals when many HIV-positive individuals lack access to primary treatment with ARVs [48]. The novel vaginal formulation described in this paper, has the potential to provide women with a non-antiretroviral approach to prevent HIV-1 infection by enhancing the protective properties of the vaginal microbiome.

## Supporting Information

S1 File Prevention of vaginal SHIV transmission in macaques by a live recombinant Lactobacillus. http://dx.doi.org/10.1038/mi.2011.30.
(PDF)

S2 File A Chinese rhesus macaque (Macaca mulatta) model for vaginal Lactobacillus colonization and live microbicide development. (PDF)

S3 File In Vivo Evaluation of Safety and Toxicity of a *Lactobacillus jensenii* Producing Modified Cyanovirin-N in a Rhesus Macaque Vaginal Challenge Model. http://dx.doi.org/10.1371/journal.pone.0078817
(PDF)
