# Supplementary material for: Robust Vaginal Colonization of Macaques with a Novel Vaginally Disintegrating Tablet Containing a Live Biotherapeutic Product to Prevent HIV Infection in Women
Source: PLoS One. 2015 Apr 14;10(4):e0122730. doi: 10.1371/journal.pone.0122730 (PMC4397015; doi:10.1371/journal.pone.0122730)
Supplement: S2 File — (PDF) [file pone.0122730.s002.pdf]

J Med Primatol doi:10.1111/j.1600-0684.2008.00316.x

ORIGINAL ARTICLE

A Chinese rhesus macaque (*Macaca mulatta*) model for vaginal *Lactobacillus* colonization and live microbicide development

Rosa R. Yu[1], Andrew T. Cheng[1], Laurel A. Lagenaur[1], Wenjun Huang[1], Deborah E. Weiss[2], Jim Treece[2], Brigitte E. Sanders-Beer[3,*], Dean H. Hamer[4], Peter P. Lee[1], Qiang Xu[1] & Yang Liu[1]

1 Osel, Inc., Santa Clara, CA, USA
2 Advanced BioScience Laboratories, Inc., Kensington, MD, USA
3 Southern Research Institute, Frederick, MD, USA
4 National Cancer Institute, National Institute of Health, Bethesda, MD, USA

Keywords
bacterial colonization – Chinese rhesus macaques – *Lactobacillus johnsonii* – live microbial microbicide – vaginal microflora

Correspondence
Qiang Xu, PhD, Osel, Inc., 4008 Burton Dr., Santa Clara, CA 95054, USA.
Tel.: +1 408 9860012 ext. 205;
fax: +1 408 9860019;
e-mail: qxu@oselinc.com
Yang Liu, PhD, Osel, Inc., 4008 Burton Dr., Santa Clara, CA 95054, USA.
Tel.: +1 408 9860012 ext. 209;
fax: +1 408 9860019;
e-mail: yliu@oselinc.com

*Present address: BIOQUAL, Inc., 9600 Medical Center Dr., Rockville, MD 20850, USA.

Accepted July 31, 2008.

Abstract

Background We sought to establish a nonhuman primate model of vaginal *Lactobacillus* colonization suitable for evaluating live microbial microbicide candidates.
Methods Vaginal and rectal microflora in Chinese rhesus macaques (*Macaca mulatta*) were analyzed, with cultivable bacteria identified by 16S rRNA gene sequencing. Live lactobacilli were intravaginally administered to evaluate bacterial colonization.
Results Chinese rhesus macaques harbored abundant vaginal *Lactobacillus*, with *Lactobacillus johnsonii* as the predominant species. Like humans, most examined macaques harbored only one vaginal *Lactobacillus* species. Vaginal and rectal *Lactobacillus* isolates from the same animal exhibited different genetic and biochemical profiles. Vaginal *Lactobacillus* was cleared by a vaginal suppository of azithromycin, and endogenous *L. johnsonii* was subsequently restored by intravaginal inoculation. Importantly, prolonged colonization of a human vaginal *Lactobacillus jensenii* was established in these animals.
Conclusions The Chinese rhesus macaque harbors vaginal *Lactobacillus* and is a potentially useful model to support the pre-clinical evaluation of *Lactobacillus*-based topical microbicides.

Introduction

Human mucosal surfaces such as the non-keratinized epithelium of the vagina are colonized by commensal flora [1, 11, 21, 50]. The vagina, together with its microflora, constitutes a dynamic ecosystem with important host defense mechanisms that promote reproductive health in women. In healthy women of childbearing age, the vaginal flora is dominated by lactobacilli (10^7–10^9 CFU per gram of fluid) [41]. These facultative anaerobes metabolize glucose to lactic acid, contributing to the maintenance of a low vaginal pH (3.6–4.5) that accounts for a major part of the non-specific defense to reduce the risk of acquiring sexually transmitted diseases [5]. Depletion of vaginal lactobacilli is associated with establishment of opportunistic bacterial infections [14] and an increased risk of acquiring human immunodeficiency virus (HIV) and herpes simplex virus type 2 (HSV-2) [9, 10, 30, 40, 43]. Consequently, there has been considerable interest in exploring an ecologic approach to re-populate the vagina with lactobacilli, termed *Lactobacillus* replacement therapy. One unique biologic approach being pursued is the use of a 'live' recombinant human

 © 2008 The Authors
Journal compilation © 2008 Blackwell Munksgaard

vaginal *Lactobacillus* as a self-renewable topical microbicide against vaginal HIV transmission [8, 27, 28].

Some smaller animals, including mice, rats, dogs, hamsters, and guinea pigs are reported to harbor low numbers of vaginal lactobacilli [34, 38]. Apparently, lactobacilli are not the dominant species in the vaginal flora as in the case of reproductive-age women. In addition, these smaller animals have an estrous cycle, so their reproductive physiology as well as anatomy is also very different from that of humans [34, 38]. Thus, selection of a relevant animal model that is closely related to humans and sensitive to mucosal HIV infection is crucial. We sought to establish a nonhuman primate model that could more accurately mimic the human female lower reproductive tract. This model would help to test colonization, persistence, efficacy, and safety of a 'live' topical microbicide against mucosal transmission of pathogens in the vagina.

Among nonhuman primates, the rhesus macaque (*Macaca mulatta*) reproductive system shares many similarities to the human reproductive system. They have been widely used in simian immunodeficiency virus and simian-human immunodeficiency virus vaginal challenge models [19, 29] to test various candidate microbicides [26, 45, 47–49]. In order to develop a rhesus macaque model for evaluating *Lactobacillus*-based microbicides, we analyzed the cultivable vaginal flora over one menstrual cycle of 13 Chinese rhesus macaques that were housed at two separate facilities. We recovered abundant endogenous vaginal *Lactobacillus* species in 12 of 13 animals and identified *Lactobacillus johnsonii* as the predominant species in these macaques. Moreover, vaginal colonization of a human vaginal *Lactobacillus* strain was established in these animals. This is the first report of persistent vaginal colonization of human *Lactobacillus* in the Chinese rhesus macaque. These findings demonstrate that the female Chinese rhesus macaque is a potentially useful model to test vaginal *Lactobacillus* replacement therapy for prevention of urogenital infections and to support the pre-clinical evaluation of *Lactobacillus*-based topical microbicides.

Materials and methods

Experimental animals

Thirteen sexually mature, non-hormonal-treated female Chinese-origin rhesus macaques, ranging in age from 4 to 10 years, were individually housed at two separate AAALAC International accredited facilities in Maryland. Specifically, five monkeys were housed at Southern Research Institute and eight monkeys were housed at Advanced Bioscience Laboratories (ABL). The procedures were conducted under protocols approved by the Institutional Animal Care and Use Committee (protocol no. 348 at ABL, and protocol no. 05-05-047F at Southern Research Institute). Animals were housed, cared for and used humanely in accordance with the 'Guide for the Care and Use of Laboratory Animals' by the Institute of Laboratory Animal Resources, Commission on Life Sciences, National Research Council. The animals housed at Southern Research Institute received a high protein monkey diet 5045 (Lab Diet, St. Louis, MO, USA), various fruits and vegetables, and nutritious supplements. The animals housed at ABL received a similar diet, including a high protein monkey chow (Ralston-Purina, St. Louis, MO, USA) supplemented with fruits and healthy treats.

Sampling

Prior to sample collections or intravaginal administration of vaginal suppository of azithromycin or lactobacilli, the animals were sedated with an injectable anesthetic, ketamine hydrochloride, at a dose of 10 mg/kg. Once sedated, the animals were removed from their cage and taken to a central procedure room. Vaginal and rectal cultures were obtained with polyester-tipped swabs (Becton-Dickinson, Cockeysville, MD, USA) and transported overnight to the Microbiology Core Laboratory at Osel Inc., CA in Port-A-Cul anaerobic transport tubes (Becton-Dickinson), which preserve the viability of anaerobic and aerobic bacterial specimens [4]. Approximately 130–150 mg of vaginal fluid (containing some sloughed-off vaginal epithelial cells) was obtained with the swabs. Vaginal fluid was smeared immediately onto pH-indicator strips (EMD Chemicals Inc., Darmstadt, Germany) to estimate vaginal pH values. They typically varied from pH 4 to 8 in Chinese rhesus macaques undergoing normal menstrual cycles.

Microbiology

The microflora was analyzed by standard culture-dependent assays, with modifications of previously described methods [35]. Briefly, the vaginal and rectal swabs were removed from the transport tubes and serially diluted in saline buffer in an anaerobic chamber, Bactron IV (Sheldon Manufacturing, Cornelius, OR, USA). Serial dilutions of the bacterial samples were then plated on different agar plates and incubated anaerobically at 37°C with 5% hydrogen, 5% CO_2, and 90% nitrogen for 4–5 days, or aerobically at 37°C with 5% CO_2 for 24–48 hours. The media used for

J Med Primatol (2008) 1–12 © 2008 The Authors
Journal compilation © 2008 Blackwell Munksgaard

anaerobic culture were Brucella blood agar, phenylethyl alcohol (PEA) agar with 5% sheep blood, laked blood with kanamycin and vancomycin agar, bacteroides bile esculin agar (Anaerobe Systems, Morgan Hill, CA, USA), and Mann Rogosa Sharpe agar (MRS) (EMD Chemicals Inc., Gibbstown, NJ, USA). The media used for aerobic culture were tryptic soy agar with 5% sheep blood, PEA with 5% sheep blood, MacConkey agar, Candida isolation agar (Hardy Diagnostics, Santa Maria, CA, USA), MRS agar and Rogosa agar (Becton, Dickinson and Co., Franklin Lakes, NJ, USA). Cultures on the primary plates were screened, semi-quantitated, and sub-cultured for isolation of pure cultures. They were then examined for pigment production and hemolysis of blood agar. Additional Gram stain and biochemical tests (e.g. catalase, oxidase, urease and indole production) were also performed for identification of the bacterial isolates.

Bacterial identification by 16S ribosomal RNA (rRNA) gene sequencing

Bacterial identification to the species level was performed using 16S rRNA gene sequencing. A single colony of bacteria was lysed in 100 μl of 'PrepMan Ultra' Sample Preparation Reagent (Applied Biosystems, Foster City, CA, USA) to prepare a DNA template for polymerase chain reaction (PCR) amplification. 16S rDNA fragments ($\sim$900 base pairs) were amplified with universal bacterial primers 8f (5′-AGA GTT TGA TCC TGG CTC AG-3′) and 926r (5′-CCG TCA ATT CCT TTR AGT TT-3′, R = A/G) [11], purified with ExoSAP-IT (USB, Cleveland, OH, USA), and sequenced with a primer 519r (5′-GWA TTA CCG CGG CKG CTG-3′, W = A/T, K = G/T) [32]. Resulting sequences were subjected to nucleotide–nucleotide BLAST (blastn) in comparison to known 16S rRNA genes in the public databases to identify the species of *Lactobacillus*. A species was assigned to an isolate when it shared 98% or higher identity to known genes. For construction of a phylogenetic tree, full-length 16S rRNA genes of *Lactobacillus* were PCR amplified using primers 8f and 1492r (5′-TAC GGY TAC CTT GTT ACG ACT T-3′, Y = C/T) [21]. The resulting PCR products were cloned into pGEM-T Easy Vector System (Promega, Madison, WI, USA) and sequenced with universal primers SP6 and T7.

Assay for detection of hydrogen peroxide production by *Lactobacillus* isolates

Hydrogen peroxide (H_2O_2) production by *Lactobacillus* strains was tested on MRS agar supplemented with tetramethylbenzidine and horseradish peroxidase (Sigma-Aldrich, St. Louis, MO, USA) [42]. Plates were incubated anaerobically at 37°C for 24 hours and then exposed to ambient air at room temperature. Colonies were observed for color development (from white to blue, indicating H_2O_2 production) for 30 min. H_2O_2 production was ranked as $+++$, $++$, and $+$, if blue color developed in less than 10, 20, and 30 min, respectively. H_2O_2 production was ranked as negative if colonies remained white within 30 min.

Carbohydrate fermentation by *Lactobacillus* isolates

Phenotypic diversity among *Lactobacillus* isolates to ferment carbohydrates was evaluated using API 50 CH carbohydrate fermentation strips (bioMerieux, Inc., Marcy-I'Etoile, France) [6]. Pure *Lactobacillus* colonies that were grown anaerobically on MRS agar plates for 24 hours were harvested with a sterile swab, and resuspended in an API 50 CHL medium to turbidity equal to or greater than 0.5 McFarland. Strips were incubated anaerobically for 24–48 hours and results were recorded.

Repetitive extragenic palindromic (Rep)-PCR

Chromosomal DNA of *Lactobacillus* culture at early stationary phase was isolated by using the DNeasy Blood and Tissue kit (Qiagen, Valencia, CA, USA). An aliquot of chromosomal DNA was used for Rep-PCR using primers Rep-R (5′-III NCG NCG NCA TCN GGC-3′, I = inosine, N = C/G/A/T) and Rep-L (5′-NCG NCT TAT CNG GCC TAC-3′, N = C/G/A/T) in the PCR mixtures [2]. PCR was performed with a PTC-200 model thermal cycler (MJ Research, Waltham, MA, USA). The PCR reaction was initiated by incubating the reaction mixture at 95°C for 7 min to activate the Taq DNA polymerase (Invitrogen, Carlsbad, CA, USA), followed by 35 cycles of 90°C for 30 s, 40°C for 1 min, and 65°C for 8 min. The reaction was terminated with an extension step of 65°C for 16 min. An aliquot of each reaction mixture was resolved on a 1% agarose gel at 30 V for 18 hours.

In vitro testing of antibiotic susceptibility of vaginal *Lactobacillus* isolates

A broth microdilution method [33] was used to test the minimum inhibitory concentrations (MICs) of antibiotics for vaginal *Lactobacillus* isolates.

Clearance of vaginal *Lactobacillus* by vaginal suppository of azithromycin

Fatty acid-based suppositories containing 200 mg of azithromycin were compounded by Foer's Pharmacy (Bethesda, MD, USA) and administered intravaginally once a day for five consecutive days by digital manipulation to sedated animals.

Intravaginal administration of *L. johnsonii* and human vaginal isolate of *L. jensenii* to Chinese rhesus macaques

Endogenous vaginal isolates of *L. johnsonii* isolated from two Chinese rhesus macaques and a human vaginal isolate of *L. jensenii* 1153 [28] were grown overnight to late log/early stationary phase in MRS broth. The bacterial cells were centrifuged and washed with PBS (pH 7.0). Approximately 10^9 CFU of *Lactobacillus* cells were mixed in 1 ml of fresh MRS medium and 2 ml of 2.7% hydroxyethyl cellulose (HEC) solution [44], and inoculated intravaginally into the sedated macaques for 7 consecutive days for *L. jensenii* 1153 [28] or every other day for three times for *L. johnsonii* recovered from rhesus macaques. Sedated animals were closely monitored by veterinary staff.

Results

Lactobacillus was isolated among endogenous vaginal microflora of Chinese rhesus macaques

The vaginal microflora of nine naïve Chinese rhesus macaques that have not been treated with antibiotics was analyzed from samples collected at multiple time points over one menstrual cycle (36–46 days). Cultivable bacterial species isolated from these animals over the course of the studies are listed in Table 1. The most common bacterial species isolated were *Enterococcus* and *Staphylococcus* species (100%). Surprisingly, *Lactobacillus* was the next most common bacterial species isolated from these animals (89%). Six out of nine animals harbored H_2O_2-producing *Lactobacillus* species, while four out of nine animals harbored non-H_2O_2-producing *Lactobacillus* species. Among the anaerobes, Gram-positive cocci *Peptoniphilus* sp., *Peptostreptococcus* sp., and *Anaerococcus* sp., and Gram-negative rods *Bacteroides* sp. and *Porphyromonas* sp. were the most frequently and the most prevalent ones isolated. Yeast was not isolated from any of the animals.

Table 1 Vaginal microflora of Chinese rhesus macaques

Organism	Number of macaques harboring the organism (n = 9)
Aerobic/facultative anaerobic	
Lactobacillus sp.	8
$H_2O_2^+$	6
$H_2O_2^-$	4
Enterococcus sp.	9
Staphylococcus sp.	9
Streptococcus sp.	6
Micrococcus sp.	7
Escherichia coli	5
Proteus sp.	5
Aerococcus sp.	3
Aerosphaera sp.	1
Corynebacterium sp.	1
Citrobacter sp.	1
Facklamia sp.	1
Klebsiella sp.	1
Psychrobacter sp.	1
Vagococcus sp.	1
Weissella sp.	1
Anaerobic	
Peptoniphilus sp.	5
Peptostreptococcus sp.	3
Anaerococcus sp.	2
Finegoldia sp.	1
Micromonas micros	1
Propionibacterium sp.	2
Bifidobacterium sp.	1
Bacteroides sp.	4
Porphyromonas sp.	4
Prevotella sp.	1
Bilophila wadsworthia	1
Veillonella sp.	2

Identification and characterization of endogenous vaginal *Lactobacillus* in Chinese rhesus macaques

Lactobacillus isolated from a total of 13 Chinese rhesus macaques housed at two separate locations was analyzed. Analysis of 16S rRNA gene sequences determined that the predominant *Lactobacillus* species recovered from the vagina of 12 of 13 macaques is *L. johnsonii* (Table 2). Endogenous vaginal *Lactobacillus* was not recovered from one of the animals (ABL-5) over the course of the study. A majority of the macaques only harbored one vaginal *Lactobacillus* species, but a few of them harbored more than one. The level of cultivable lactobacilli ranged from 10^2 to 10^8 CFU per vaginal swab. Two isolates of *L. johnsonii* that had different colony morphology on the MRS agar plates were recovered from one macaque, SRI-3. When carbohydrate fermentation profiles were compared between these two vaginal

J Med Primatol (2008) 1–12 © 2008 The Authors
Journal compilation © 2008 Blackwell Munksgaard

Table 2 Characterization of endogenous vaginal and rectal *Lactobacillus* isolates recovered from Chinese rhesus macaques

Animal	Isolate	Origin of isolation	*Lactobacillus* species[1]	Carbohydrate fermentation by API 50 CH tests[2]																										$H_2O_2^3$ production
				LARA	RIB	GAL	GLU	FRU	MNE	DUL	MAN	SOR	MDG	NAG	AMY	ARB	ESC	SAL	CEL	MAL	LAC	MEL	SAC	TRE	RAF	AMD	GEN	TUR	TAG	
ABL-1	ABL-V1	Vagina	*L. johnsonii*	■	■					■	■	■	■									■			■			■		–
ABL-2	ABL-V2	Vagina	*L. johnsonii*	■	■					■	■	■	■								■							■		+++
ABL-3	ABL-V3	Vagina	*L. johnsonii*	■	■					■	■	■	■									■			■			■		+++
ABL-4	ABL-V4	Vagina	*L. johnsonii*	■	■					■	■	■	■									■			■			■	■	–
ABL-6	ABL-V6A	Vagina	*L. johnsonii*	■	■					■	■	■	■									■			■	■		■	■	–
	ABL-R6A	Rectum	*L. johnsonii*	■		■			■	■	■	■	■	■	■	■	■	■			■			■		■	■	■	■	–
	ABL-R6B	Rectum	*L. johnsonii*	■	■								■		■	■	■	■								■	■	■		+++
	ABL-V6B	Vagina	*L. murinus*	■						■	■	■	■		■	■	■	■						■		■	■	■	■	–
	ABL-R6C	Rectum	*L. murinus*							■	■	■	■														■	■		–
ABL-7	ABL-V7A	Vagina	*L. johnsonii*	■	■					■	■	■	■															■		+++
	ABL-R7A	Rectum	*L. johnsonii*	■	■					■	■	■	■									■			■			■		–
	ABL-V7B	Vagina	*L. acidophilus*	■	■					■	■	■																	■	–
	ABL-R7B	Rectum	*L. acidophilus*	■	■					■	■	■																	■	+++
	ABL-V7C	Vagina	*L. amylovorous*	■	■					■	■	■	■									■		■				■	■	+++
ABL-8	ABL-V8A	Vagina	*L. johnsonii*	■	■					■	■	■	■									■						■		+++
	ABL-R8A	Rectum	*L. johnsonii*	■	■					■	■	■									■	■						■		–
	ABL-V8B	Vagina	*L. amylovorous*	■	■					■	■	■	■		■	■		■				■		■		■		■	■	–
SRI-1	SRI-V1	Vagina	*L. johnsonii*	■	■					■	■	■	■								■	■			■			■	■	+++
	SRI-R1	Rectum	*L. johnsonii*	■	■					■	■	■	■								■	■						■		+++
SRI-2	SRI-V2	Vagina	*L. johnsonii*	■	■					■	■	■	■								■							■		–
SRI-3	SRI-V3A	Vagina	*L. johnsonii*	■	■					■	■	■	■								■	■			■			■	■	+++
	SRI-V3B	Vagina	*L. johnsonii*	■	■					■	■	■	■								■	■						■		+++
	SRI-R3	Rectum	*L. johnsonii*	■	■				■	■	■	■	■			■		■			■	■		■				■	■	+++
SRI-4	SRI-V4	Vagina	*L. johnsonii*	■	■				■	■	■	■	■								■	■			■			■		+++
	SRI-R4	Rectum	*L. johnsonii*	■	■					■	■	■	■								■	■			■			■		+++
SRI-5	SRI-V5	Vagina	*L. johnsonii*	■	■					■	■	■	■								■	■			■			■		+++
	SRI-R5	Rectum	*L. johnsonii*	■	■					■	■	■	■								■	■				■		■		+++

[1]Species of *Lactobacillus* isolates was determined by 16S rRNA gene sequencing as described in Materials and Methods.
[2]White box: positive fermentation; grey box: negative fermentation. LARA, L-arabinose; RIB, D-ribose; GAL, D-galactose; GLU, D-glucose; FRU, D-fructose; MNE, D-mannose; DUL, dulcitol; MAN, D-mannitol; SOR, D-sorbitol; MDG, methyl-αD-glucopyranoside; NAG, *N*-acetylglucosamine; AMY, amygdalin; ARB, arbutin; ESC, esculin ferric citrate; SAL, salicin; CEL, D-cellobiose; MAL, D-maltose; LAC, D-lactose; MEL, D-melibiose; SAC, D-saccharose; TRE, D-trehalose; RAF, D-raffinose; AMD, amidon (starch); GEN, gentiobiose; TUR, D-turanose; TAG, D-tagatose.
[3]+++: strong H_2O_2 production; -: no H_2O_2 production in 30 min.

L. johnsonii isolates, differences were noted in the fermentation of D-lactose, D-raffinose and D-tagatose, demonstrating that they are different strains (Table 2). Other vaginal *Lactobacillus* species were also recovered from three macaques which harbored *L. johnsonii*. *Lactobacillus murinus* was isolated from ABL-6, *L. amylovorous* was isolated from ABL-8, and *L. acidophilus* and *L. amylovorous* were recovered from ABL-7 (Table 2). However, these other *Lactobacillus* species constituted the minority, as they were recovered less consistently (at one time point only) than *L. johnsonii*. Most of the endogenous lactobacilli recovered from Chinese rhesus macaques are strong H_2O_2 producers (Table 2). Nine of 13 strains of *L. johnsonii* are strong H_2O_2 producers. For the other vaginal *Lactobacillus* species, only *L. amylovorous* from ABL-7 (ABL-V7C) is a H_2O_2 producer.

Rectal and vaginal *Lactobacillus* isolates recovered from the same macaque are uniquely different from each other

Rectal swabs were also collected from the above-mentioned nine naïve macaques (ABL 5-8 and SRI 1-5), and endogenous lactobacilli were recovered from all samples analyzed, including ABL-5 that did not harbor vaginal lactobacilli at any sampling times. *Lactobacillus johnsonii* was found in seven of the nine animals, but it is not the predominant *Lactobacillus* species in the rectum as in the case of the vagina. In the rectum, many different *Lactobacillus* species were recovered, including *Lactobacillus reuteri*, *Lactobacillus animalis*, *Lactobacillus ingluviei*, *Lactobacillus salivarius*, *Lactobacillus sobrius* and *Lactobacillus mucosae*. In most instances, more than one species of rectal lactobacilli was recovered from each individual macaque. Seven of the nine macaques harbored the same *Lactobacillus* species in both the vagina and the rectum (Table 2), but it was subsequently shown that they have different biochemical and genetic profiles.

Additional biochemical tests and chromosomal DNA fingerprinting conclusively determined that the rectal and vaginal *Lactobacillus* isolates from the same animal are uniquely different from each other (Table 2 and Fig. 1). All *L. johnsonii*, of both vaginal and rectal origins, could ferment D-glucose, D-fructose, D-maltose, and sucrose. However, a few rectal *L. johnsonii* isolates (ABL-R6A, ABL-R6B and SRI-R3) could not utilize several sugars (D-galactose, *N*-acetylglucosamine, amygdalin, arbutin, esculin ferric citrate, salicin, D-cellobiose, D-trehalose, and gentiobiose) that were fermented by all of the vaginal isolates. D-ribose, dulcitol, D-mannitol, D-sorbitol, and methyl-αD-glucopyranoside were used by some of the rectal but none of the vaginal *L. johnsonii* isolates. L-arabinose was fermented only by *L. murinus* isolated from the rectum of animal ABL-6 (ABL-R6C), and D-turanose was utilized only by the *L. acidophilus* isolates, of both vaginal and rectal origins, from ABL-7 (ABL-V7B and ABL-R7B) (Table 2).

Rep-PCR was also performed to determine the genomic fingerprint patterns of the *Lactobacillus* isolates. The fingerprint patterns were consistent in three independent rep-PCR runs, and a representative figure is shown in Fig. 1. As shown in Fig. 1, fingerprints of the vaginal isolates of *L. johnsonii* from animals ABL 1–4 are each different from one another, indicating that they are different isolates (Fig. 1A, lanes 2–5). When fingerprints of *L. johnsonii* (Fig. 1A, lanes 6–8) isolated from the same animal ABL-6 were analyzed, the band patterns were similar, but not identical, indicating again that the vaginal and rectal *Lactobacillus* isolates from the same animal are uniquely different from each other. Similar results were observed for the vaginal and rectal lactobacilli isolates from other animals: *L. johnsonii* isolates from ABL-7 (Fig. 1A, lanes 11 and 12), *L. acidophilus* isolates from ABL-7 (Fig. 1A, lanes 13 and 14), *L. johnsonii* from SRI-1 (Fig. 1B, lanes 2 and 3), *L. johnsonii* from SRI-3 (Fig. 1B, lanes 5–7), *L. johnsonii* from SRI-4 (Fig. 1B, lanes 8 and 9), and *L. johnsonii* from SRI-5 (Fig. 1B, lanes 10 and 11). Although the fingerprints of the vaginal and rectal *L. johnsonii* isolates from animal ABL-8 seemed almost identical (Fig. 1A, lanes 16 and 17), carbohydrate fermentation profiles revealed differences in the fermentation of methyl-αD-glucopyranoside and D-lactose, and they also differed in terms of H_2O_2 production (Table 2). Similarly, vaginal and rectal *L. murinus* (Fig. 1A, lanes 9 and 10) from ABL-6 also have very similar fingerprints, but they differed in the fermentation of multiple carbohydrates (Table 2). Together, carbohydrate fermentation profiles, H_2O_2 production, and genomic fingerprints revealed that the *Lactobacillus* isolates of the same species from the same animals are indeed different (Table 2 and Fig. 1).

Lactobacillus johnsonii isolated from Chinese rhesus macaques are closely related to *Lactobacillus gasseri*

Sequence data of the 1537-nucleotide region of the 16S rRNA gene of the vaginal *L. johnsonii* isolates from Chinese rhesus macaques were aligned using CLUSTALW and a phylogenetic tree was constructed showing the relationship of *L. johnsonii* and a few representative human vaginal isolates. As shown in Fig. 2, all vaginal *L. johnsonii* isolates from the 12 macaques clustered together. They also have a very close phylogenetic relationship to a human vaginal *L. johnsonii* isolate from our collection (*L. johnsonii* 135-1). The 16S rRNA

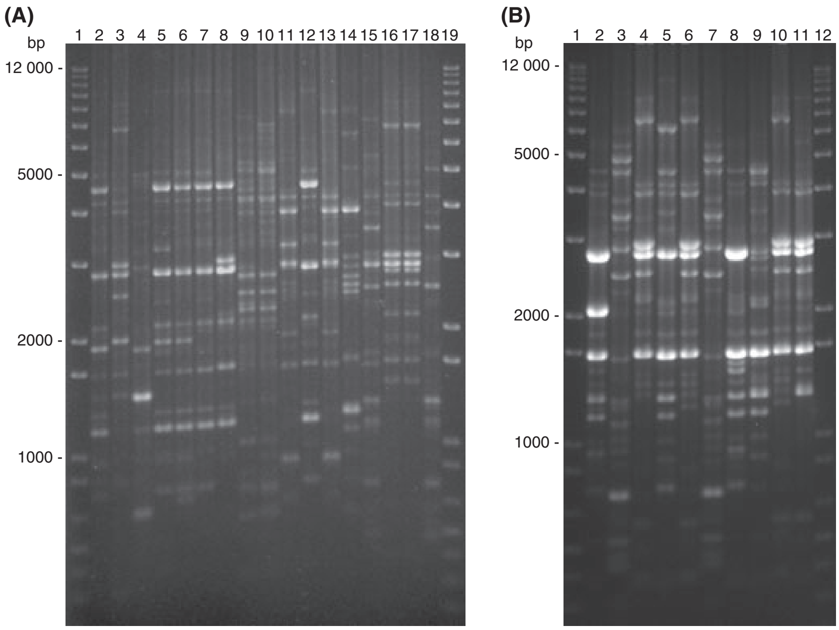

Fig. 1 Rep-PCR DNA fingerprint patterns of vaginal and rectal *Lactobacillus* strains isolated from Chinese rhesus macaques housed at (A) ABL and (B) Southern Research Institute. (A) Lanes 1 and 19, a 1-kb plus DNA ladder; lane 2, *L. johnsonii* ABL-V1; lane 3, *L. johnsonii* ABL-V2; lane 4, *L. johnsonii* ABL-V3; lane 5, *L. johnsonii* ABL-V4; lane 6, *L. johnsonii* ABL-V6A; lane 7, *L. johnsonii* ABL-R6A; lane 8, *L. johnsonii* ABL-R6B; lane 9, *L. murinus* ABL-V6B; lane 10, *L. murinus* ABL-R6C; lane 11, *L. johnsonii* ABL-V7A; lane 12, *L. johnsonii* ABL-R7A; lane 13, *L. acidophilus* ABL-V7B; lane 14, *L. acidophilus* ABL-R7B; lane 15, *L. amylovorous* ABL-V7C; lane 16, *L. johnsonii* ABL-V8A; lane 17, *L. johnsonii* ABL-R8A; lane 18, *L. amylovorous* ABL-V8B. (B) lanes 1 and 12, a 1-kb plus DNA ladder; lanes 2–11, all *L. johnsonii* isolates: lane 2, SRI-V1; lane 3, SRI-R1; lane 4, SRI-V2; lane 5, SRI-V3A; lane 6, SRI-V3B; lane 7, SRI-R3; lane 8, SRI-V4; lane 9, SRI-R4, lane 10, SRI-V5; lane 11, SRI-R5. bp: base pair of DNA fragments.

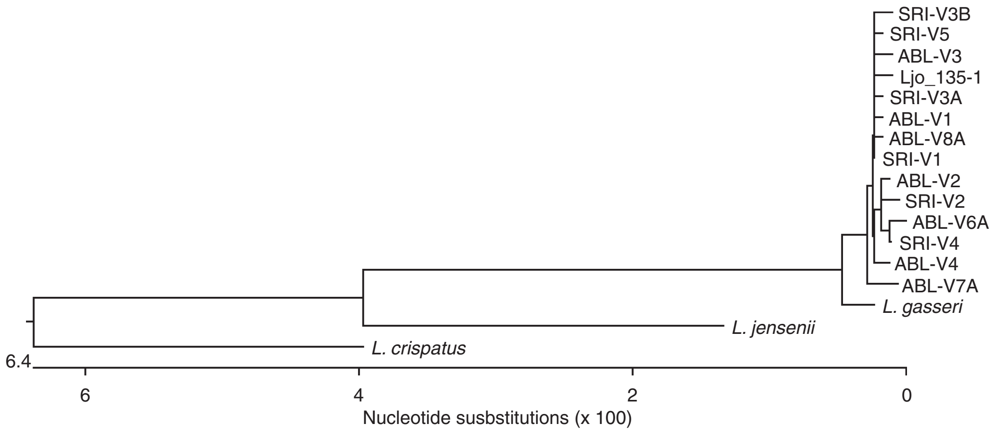

Fig. 2 Phylogenetic tree showing the relationship based on the analysis of a 1537-nucleotide region of 16S rRNA genes of vaginal *Lactobacillus johnsonii* from Chinese rhesus macaques to some representative human vaginal *Lactobacillus* species. The tree was constructed using the CLUSTALW program. Ljo_135-1 is a human vaginal isolate of *L. johnsonii*.

gene-based phylogenetic tree also showed that *L. johnsonii* has a closer relationship to human vaginal strains of *L. gasseri* than to *Lactobacillus crispatus* or *L. jensenii*.

Dynamic nature of endogenous vaginal *L. johnsonii* in Chinese rhesus macaques

In order to understand the dynamic nature of vaginal *Lactobacillus* flora over menstrual cycles, the level of endogenous vaginal lactobacilli was monitored at four time points over a period of 36 days in five Chinese rhesus macaques housed at Southern Research Institute. The macaques did not receive hormonal treatment, so their menstrual cycles were not synchronized. On average, between 10^5 and 10^7 CFU per vaginal swab of *L. johnsonii* was recovered in all macaques. For animal SRI-5, lactobacilli were not recovered at two time points (days 1 and 22). This may be because the amount of lactobacilli present at those time points was below the detection level of our microbiologic techniques. It was also noted that the level of endogenous lactobacilli dropped precipitously (to $\sim 10^4$) during or immediately after menses (Fig. 3). For other non-lactobacilli endogenous bacteria, a similar pattern of changes was also observed. The average level of other endogenous microflora recovered was also about 10^6 to 10^7 CFU per vaginal swab, and less variety and lower quantity of each bacterial species was recovered during or immediately after menses (data not shown).

Colonization of a human vaginal isolate of *L. jensenii* 1153 in Chinese rhesus macaques

To test the potential use of Chinese rhesus macaques as a model for studying colonization of human vaginal

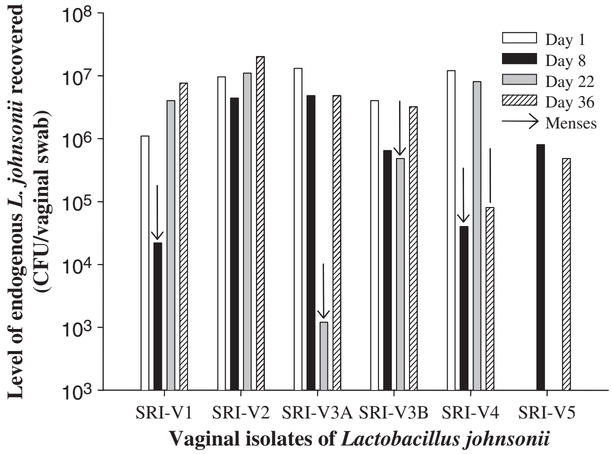

Fig. 3 Dynamic nature of endogenous vaginal *Lactobacillus johnsonii* in Chinese rhesus macaques housed at Southern Research Institute. Vaginal swabs were collected at multiple time points over one menstrual cycle, and the level of endogenous *L. johnsonii* recovered from each animal was studied. Levels of endogenous lactobacilli varied from 10^3 to 10^7 per swab during the cycle. Arrows indicated sample collection immediately (one day) after menses (animal SRI-1) or during menses (animals SRI-3 and SRI-4). Macaques were not synchronized for their menstrual cycles.

Lactobacillus, the macaques were inoculated with a human vaginal isolate of *L. jensenii* 1153 for seven consecutive days. Bacterial concentrations of 10^6 and 10^5 CFU per vaginal swab were recovered 1 and 5 days after final bacterial inoculation, respectively (Fig. 4A). *Lactobacillus jensenii* 1153 forms small smooth colonies on the MRS agar plates, which can be differentiated from the large rough colonies of the endogenous *L. johnsonii*. *L. jensenii* isolates were further confirmed by 16S rRNA gene sequencing. In

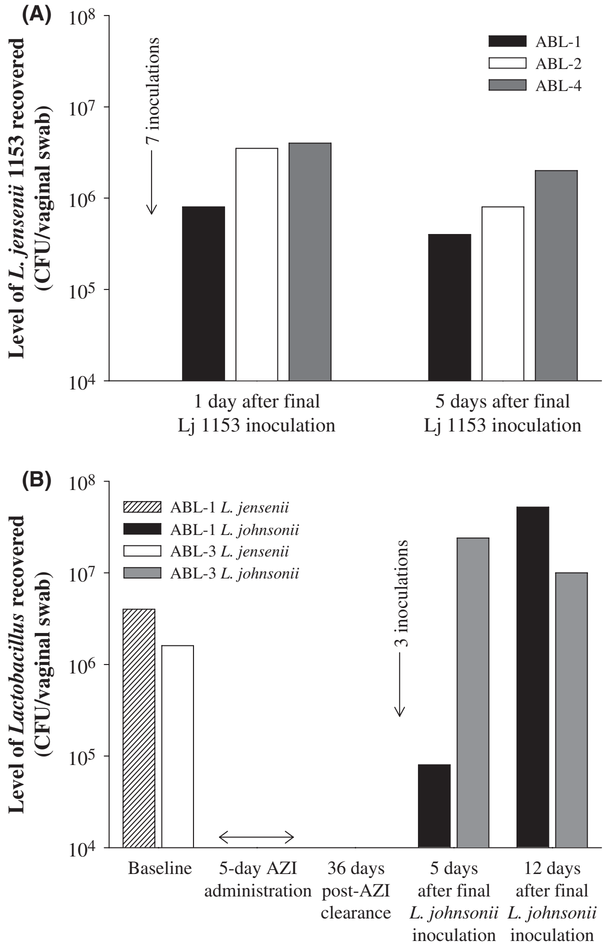

Fig. 4 Vaginal colonization of *Lactobacillus* in Chinese rhesus macaques. (A) 7-day inoculation of a human vaginal isolate of *L. jensenii* 1153 in three Chinese rhesus macaques (ABL-1, ABL-2 and ABL-4). Level of *Lactobacillus jensenii* recovered was analyzed for vaginal swabs collected at 1 and 5 days after the final bacterial inoculation. Lj 1153, *L. jensenii* 1153; 7 inoculations, 7-day inoculation of *L. jensenii* 1153. (B) Clearance of vaginal *Lactobacillus* by a 5-day administration of vaginal suppository of azithromycin, followed by restoration of *L. johnsonii* in two Chinese rhesus macaques (ABL-1 and ABL-3). Baseline level of vaginal *Lactobacillus* was analyzed before azithromycin administration. Level of *Lactobacillus* was monitored for 36 days after the final azithromycin administration. *Lactobacillus johnsonii* previously isolated from individual macaques was reintroduced intravaginally every other day for three times into each of the two macaques. Level of *L. johnsonii* was analyzed for vaginal swabs collected at 5 and 12 days after the final bacterial inoculation. AZI, vaginal suppository of azithromycin; 3 inoculations, 3 inoculations (every other day) of *L. johnsonii*.

one of the macaques, 9×10^6 CFU per vaginal swab of *L. jensenii* was recovered 26 days after the final bacterial inoculation (data not shown). Apparently, the introduction of the human vaginal *Lactobacillus* isolate has no significant impact on the diversity or quantity of the endogenous vaginal flora of the macaques (data not shown).

Clearance of vaginal *Lactobacillus* and restoration of *L. johnsonii* in Chinese rhesus macaques

A human vaginal isolate of *L. jensenii* 1153 strain and the *Lactobacillus* isolates recovered from Chinese rhesus macaques were sensitive to erythromycin and azithromycin, an erythromycin derivative (data not shown). We chose to use azithromycin as a vaginal suppository to clear vaginal *Lactobacillus* as it is acid stable (which is important in a healthy vaginal setting) and is better tolerated by animals and humans [17, 24]. By administering a vaginal suppository of azithromycin for 5 consecutive days, the exogenous human vaginal lactobacilli previously administered to the animals were readily cleared from the vaginal flora of Chinese rhesus macaques (Fig. 4B). In addition, no lactobacilli were recovered up to at least 36 days after the final azithromycin administration (Fig. 4B). Furthermore, endogenous vaginal *L. johnsonii* could be restored in these macaques. Respective isolates of *L. johnsonii* from individual macaques were reintroduced intravaginally into each of the two macaques. Approximately 10^7 CFU per vaginal swab of *L. johnsonii* were recovered 12 days after the final bacterial inoculation (Fig. 4B), demonstrating that endogenous *L. johnsonii* was restored in the Chinese rhesus macaques. On the contrary, no human vaginal *Lactobacillus* isolates were recovered from the 'reconstructed' microflora after azithromycin treatment, showing effective clearance of the pre-existing lactobacilli by the antibiotic.

Discussion

Macaques are commonly used for evaluation of the safety and efficacy of topical vaginal microbicides for preventing sexually transmitted diseases in humans [26, 45, 47–49]. The similarities between the reproductive anatomy and physiology of human and rhesus macaques [36] prompted us to establish a nonhuman primate model suitable to evaluate the colonization of our *Lactobacillus*-based topical microbicide candidates. This study describes the use of the Chinese rhesus macaque as a potential model for persistent vaginal colonization of human *Lactobacillus*, and for pre-clinical evaluation of *Lactobacillus*-based topical microbicides. Our data demonstrated that Chinese rhesus macaques naturally harbor endogenous *L. johnsonii* among their vaginal flora.

Although several small animal models (such as rabbits, mice and rats) could possibly support transient

vaginal colonization of *Lactobacillus* [7, 31, 34], they are not suitable or relevant for our purpose. All of these small animals require treatment with estrogen, and the profiles of their indigenous microflora show no resemblance to the microflora of humans [22, 34]. Rabbits in particular are not suitable, as it has been reported that laboratory rabbits are usually in precoital status. They do not undergo estrous cycle stages and have little mucous secretion before mating. Vaginal environments with poor mucus secretion are inadequate for bacterial proliferation. Thus, the vaginal environment in precoital rabbits was suggested to be comparable to that during diestrus or anestrus in other animal species [34]. We found that diestrus does not support *Lactobacillus* colonization in a mouse model [28]. It also appears that the predominance of lactobacilli and low vaginal pH are distinct characteristics of the human vaginal microflora [5, 20]. However, small animals have shown minimal numbers of vaginal lactobacilli and neutral vaginal pH [22, 31, 34]. In addition, anaerobic bacteria were the predominant flora in vagina of humans, and the total number of bacteria was higher than that in small laboratory animal models [18, 34]. The lack of similarity prompted us to look for a primate model that better mimics the human vaginal environment for the testing of live topical vaginal microbicides.

The normal vaginal flora we identified from the Chinese rhesus macaques in this study was generally similar to the findings of Doyle et al. [12] and Scorpio et al. [39] (Table 1). However, there are several major differences in the findings of these studies. First, we isolated and identified vaginal *Lactobacillus* in 12 of 13 of the animals we studied, and some of these macaques harbored more than one species of *Lactobacillus* over the course of the study. *Lactobacillus johnsonii* was the predominant endogenous vaginal *Lactobacillus* species we recovered, and it was isolated from animals housed at both ABL and Southern Research Institute. On the contrary, Doyle et al. did not find any lactobacilli in 37 rhesus macaques, and instead, reported *Gardnerella*-like or *Gardnerella*-probable organisms in about one-third of the animals, and *Mobiluncus curtisii* in half of the animals they studied. Although sampling time and bacterial identification methods were different in these two studies, it is difficult to determine whether these reasons alone contributed to the discrepancies. It was also unclear whether naïve animals were used in the studies of Doyle et al. For instance, endogenous vaginal *Lactobacillus* is sensitive to certain antibiotic treatments, as evidenced in our studies. Although Scorpio et al. reported *Lactobacillus* spp. as one of the most commonly cultured bacteria from the 11 Chinese rhesus macaques in their study, it is unclear what species these lactobacilli are and how many animals harbor them [39].

In healthy women of childbearing age, the vaginal flora is dominated by *Lactobacillus* (10^7–10^9 CFU per gram of fluid) [41]. The species of *Lactobacillus* most commonly isolated from the reproductive tracts of healthy women worldwide belong to *L. acidophilus* complex that has been subdivided into six distinct species: *L. acidophilus*, *L. crispatus*, *L. gasseri*, *L. gallinarum*, *L. amylovorous*, and *L. johnsonii* [15, 23, 25, 50]. With the advancement of molecular biology techniques, it was revealed that *L. gasseri, L. jensenii, L. crispatus*, and *Lactobacillus iners* are the most commonly isolated endogenous *Lactobacillus* species from healthy women at child-bearing age [1, 16, 46, 50]. It is interesting that three of the four endogenous *Lactobacillus* species, *L. johnsonii*, *L. amylovorous*, and *L. acidophilus* that were recovered from Chinese rhesus macaques belong to the *L. acidophilus* complex, with *L. johnsonii* as the most frequently isolated and the most prevalent vaginal *Lactobacillus* species in these macaques. *Lactobacillus johnsonii* is more closely related to *L. gasseri* phylogenetically than to *L. jensenii* or *L. crispatus* (Fig. 2 and [37]). The finding of *L. acidophilus* complex in the vagina of Chinese rhesus macaques may indicate that the microenvironmental niche for the growth and persistence of vaginal lactobacilli is similar in human and macaque. Indeed, the carbohydrate utilization profiles of the *L. johnsonii* isolates from macaques (Table 2) and the human vaginal *Lactobacillus* isolates we collected are very similar (unpublished data). This may explain why the human vaginal *L. jensenii* isolates could colonize in the vagina of macaques. In a different study, we also isolated vaginal *L. johnsonii* from seven out of nine rhesus macaques of Indian-origin and successfully colonized human vaginal *Lactobacillus* in these animals (unpublished data), which further supports our hypothesis that the similarity between rhesus macaque and human vaginal microflora makes these animals a relevant model for assessing the safety, efficacy and distribution of *Lactobacillus*-based vaginal microbicides.

Antonio et al. suggested that *Lactobacillus* from the rectum could be a potential source to seed the vagina in women [3]. However, we found that although the same species of *Lactobacillus* could be isolated from both the vagina and rectum of the macaques, they are not identical, suggesting that the rectal microflora may not be the reservoir for the endogenous vaginal *Lactobacillus*. While we could not rule out that our analysis may miss a minor *Lactobacillus* present at a very low quantity in the vaginal swabs, the origin of endogenous vaginal *Lactobacillus* remains to be elucidated.

In this study, we demonstrated the presence of abundant endogenous vaginal *Lactobacillus* in rhesus macaques that have been widely used to test various candidate microbicides. In this animal model, endogenous vaginal *Lactobacillus* microflora could serve as a complimentary marker for preclinical toxicity evaluation of potential vaginal microbicide candidates. In addition, this is a potentially useful model for vaginal *Lactobacillus* colonization to support the pre-clinical evaluation of *Lactobacillus*-based topical microbicides. Indeed, we have successfully colonized a genetically modified *L. jensenii* strain that produces a heterologous protein for at least 87 days in this rhesus macaque model (unpublished data). We also determined that it is unnecessary to clear the endogenous flora for the colonization of exogenous human *Lactobacillus* in Chinese rhesus macaques (unpublished data). The decrease in the quantity of vaginal *L. johnsonii* recovered during or immediately after menses in macaques was similar to what has been reported with humans [13]. Therefore, immediately after menses should be the best time to intravaginally administer ecologically appropriate *Lactobacillus*.

Acknowledgments

This work was supported in part by NIH Integrated Preclinical/Clinical Program for Topical Microbicides U19AI60615 and NIH Partnerships for Topical Microbicides U01AI066708. The authors thank Lorna K. Rabe at Magee-Womens Research Institute for technical advice in quantitative microbiology, Dr. Dusty Rhodes and Dawn Golightly at Southern Research Institute for their guidance and assistance in macaque studies, and Dr. Xiaowen Liu for critical reading of this manuscript.

References

1 Antonio MA, Hawes SE, Hillier SL: The identification of vaginal *Lactobacillus* species and the demographic and microbiologic characteristics of women colonized by these species. *J Infect Dis* 1999; **180**:1950–6.

2 Antonio MA, Hillier SL: DNA fingerprinting of *Lactobacillus crispatus* strain CTV-05 by repetitive element sequence-based PCR analysis in a pilot study of vaginal colonization. *J Clin Microbiol* 2003; **41**:1881–7.

3 Antonio MA, Rabe LK, Hillier SL: Colonization of the rectum by *Lactobacillus* species and decreased risk of bacterial vaginosis. *J Infect Dis* 2005; **192**:394–8.

4 Baron EJ, Vaisanen ML, McTeague M, Strong CA, Norman D, Finegold SM: Comparison of the Accu-CulShure system and a swab placed in a B-D Port-a-Cul tube for specimen collection and transport. *Clin Infect Dis* 1993; **16**:S325–7.

5 Boskey ER, Cone RA, Whaley KJ, Moench TR: Origins of vaginal acidity: high D/L lactate ratio is consistent with bacteria being the primary source. *Hum Reprod* 2001; **16**:1809–13.

6 Boyd MA, Antonio MA, Hillier SL: Comparison of API 50 CH strips to whole-chromosomal DNA probes for identification of *Lactobacillus* species. *J Clin Microbiol* 2005; **43**:5309–11.

7 Catalone BJ, Kish-Catalone TM, Budgeon LR, Neely EB, Ferguson M, Krebs FC, Howett MK, Labib M, Rando R, Wigdahl B: Mouse model of cervicovaginal toxicity and inflammation for preclinical evaluation of topical vaginal microbicides. *Antimicrob Agents Chemother* 2004; **48**:1837–47.

8 Chang TL, Chang CH, Simpson DA, Xu Q, Martin PK, Lagenaur LA, Schoolnik GK, Ho DD, Hillier SL, Holodniy M, Lewicki JA, Lee PP: Inhibition of HIV infectivity by a natural human isolate of *Lactobacillus jensenii* engineered to express functional two-domain CD4. *Proc Natl Acad Sci USA* 2003; **100**:11672–7.

9 Cherpes TL, Melan MA, Kant JA, Cosentino LA, Meyn LA, Hillier SL: Genital tract shedding of herpes simplex virus type 2 in women: effects of hormonal contraception, bacterial vaginosis, and vaginal group B streptococcus colonization. *Clin Infect Dis* 2005; **40**:1422–8.

10 Cohn MA, Frankel SS, Rugpao S, Young MA, Willett G, Tovanabutra S, Khamboonruang C, VanCott T, Bhoopat L, Barrick S, Fox C, Quinn TC, Vahey M, Nelson KE, Weissman D: Chronic inflammation with increased human immunodeficiency virus (HIV) RNA expression in the vaginal epithelium of HIV-infected Thai women. *J Infect Dis* 2001; **184**:410–7.

11 Coolen MJ, Post E, Davis CC, Forney LJ: Characterization of microbial communities found in the human vagina by analysis of terminal restriction fragment length polymorphisms of 16S rRNA genes. *Appl Environ Microbiol* 2005; **71**:8729–37.

12 Doyle L, Young CL, Jang SS, Hillier SL: Normal vaginal aerobic and anaerobic bacterial flora of the rhesus macaque (*Macaca mulatta*). *J Med Primatol* 1991; **20**:409–13.

13 Eschenbach DA, Thwin SS, Patton DL, Hooton TM, Stapleton AE, Agnew K, Winter C, Meier A, Stamm WE: Influence of the normal menstrual cycle on vaginal tissue, discharge, and microflora. *Clin Infect Dis* 2000; **30**:901–7.

14 Fredricks DN, Fiedler TL, Marrazzo JM: Molecular identification of bacteria associated with bacterial vaginosis. *N Engl J Med* 2005; **353**:1899–911.

15 Fujisawa T, Benno Y, Yaeshima T, Mitsuoka T: Taxonomic study of the *Lactobacillus acidophilus* group,

 © 2008 The Authors
Journal compilation © 2008 Blackwell Munksgaard

with recognition of *Lactobacillus gallinarum* sp. nov. and *Lactobacillus johnsonii* sp. nov. and synonymy of *Lactobacillus acidophilus* group A3 (Johnson et al. 1980) with the type strain of *Lactobacillus amylovorus* (Nakamura 1981). *Int J Syst Bacteriol* 1992; **42**:487–91.
16 Giorgi A, Torriani S, Dellaglio F, Bo G, Stola E, Bernuzzi L: Identification of vaginal lactobacilli from asymptomatic women. *Microbiologica* 1987; **10**:377–84.
17 Girard AE, Girard D, English AR, Gootz TD, Cimochowski CR, Faiella JA, Haskell SL, Retsema JA: Pharmacokinetic and *in vivo* studies with azithromycin (CP-62,993), a new macrolide with an extended half-life and excellent tissue distribution. *Antimicrob Agents Chemother* 1987; **31**:1948–54.
18 Hammann R, Kronibus A, Lang N, Werner H: Quantitative studies on the vaginal flora of asymptomatic women and patients with vaginitis and vaginosis. *Zentralbl Bakteriol Mikrobiol Hyg [A]* 1987; **265**:451–61.
19 Harouse JM, Gettie A, Eshetu T, Tan RC, Bohm R, Blanchard J, Baskin G, Cheng-Mayer C: Mucosal transmission and induction of simian AIDS by CCR5-specific simian/human immunodeficiency virus SHIV (SF162P3). *J Virol* 2001; **75**:1990–5.
20 Hillier SL, Krohn MA, Rabe LK, Klebanoff SJ, Eschenbach DA: The normal vaginal flora, H_2O_2-producing lactobacilli, and bacterial vaginosis in pregnant women. *Clin Infect Dis* 1993; **16**:S273–81.
21 Hyman RW, Fukushima M, Diamond L, Kumm J, Giudice LC, Davis RW: Microbes on the human vaginal epithelium. *Proc Natl Acad Sci USA* 2005; **102**:7952–7.
22 Jacques M, Olson ME, Crichlow AM, Osborne AD, Costerton JW: The normal microflora of the female rabbit's genital tract. *Can J Vet Res* 1986; **50**:272–4.
23 Johnson JL, Phelps CF, Cummins CS, London J, Gasser F: Taxonomy of the *Lactobacillus acidophilus* group. *Int J Syst Bacteriol* 1980; **30**:53–68.
24 Langley JM, Halperin SA, Boucher FD, Smith B: Azithromycin is as effective as and better tolerated than erythromycin estolate for the treatment of pertussis. *Pediatrics* 2004; **114**:e96–101.
25 Lauer E, Helming C, Kandler O: Heterogeneity of the species *Lactobacillus acidophilus* (Moro) Hansen and Moquot as revealed by biochemical characteristics and DNA–DNA hybridization. *Zentralbl Bakteriol Mikrobiol Hyg [A]* 1980; **1**:150–68.
26 Lederman MM, Veazey RS, Offord R, Mosier DE, Dufour J, Mefford M, Piatak M Jr, Lifson JD, Salkowitz JR, Rodriguez B, Blauvelt A, Hartley O: Prevention of vaginal SHIV transmission in rhesus macaques through inhibition of CCR5. *Science* 2004; **306**:485–7.
27 Liu X, Lagenaur LA, Lee PP, Xu Q: Engineering human vaginal *Lactobacillus* to surface expression of two-domain CD4. *Appl Environ Microbiol* 2008; **74**:4626–35.
28 Liu X, Lagenaur LA, Simpson DA, Essenmacher KP, Frazier-Parker CL, Liu Y, Tsai D, Rao SS, Hamer DH, Parks TP, Lee PP, Xu Q: Engineered vaginal lactobacillus strain for mucosal delivery of the human immunodeficiency virus inhibitor cyanovirin-N. *Antimicrob Agents Chemother* 2006; **50**:3250–9.
29 Lu Y, Brosio P, Lafaile M, Li J, Collman RG, Sodroski J, Miller CJ: Vaginal transmission of chimeric simian/human immunodeficiency viruses in rhesus macaques. *J Virol* 1996; **70**:3045–50.
30 Martin HL, Richardson BA, Nyange PM, Lavreys L, Hillier SL, Chohan B, Mandaliya K, Ndinya-Achola JO, Bwayo J, Kreiss J: Vaginal lactobacilli, microbial flora, and risk of human immunodeficiency virus type 1 and sexually transmitted disease acquisition. *J Infect Dis* 1999; **180**:1863–8.
31 Meysick KC, Garber GE: Interactions between *Trichomonas vaginalis* and vaginal flora in a mouse model. *J Parasitol* 1992; **78**:157–60.
32 Munson MA, Nedwell DB, Embley TM: Phylogenetic diversity of Archaea in sediment samples from a coastal salt marsh. *Appl Environ Microbiol* 1997; **63**:4729–33.
33 National Committee for Clinical Laboratory Standards: Methods for Dilution Antimicrobial Susceptibility Tests for Bacteria that Grow Aerobically, 5th edn. Approved Standard M7-A5. Wayne, PA, USA: NCCLS, 2000.
34 Noguchi K, Tsukumi K, Urano T: Qualitative and quantitative differences in normal vaginal flora of conventionally reared mice, rats, hamsters, rabbits, and dogs. *Comp Med* 2003; **53**:404–12.
35 Patton DL, Sweeney YC, Rabe LK, Hillier SL: The vaginal microflora of pig-tailed macaques and the effects of chlorhexidine and benzalkonium on this ecosystem. *Sex Transm Dis* 1996; **23**:489–93.
36 Poonia B, Walter L, Dufour J, Harrison R, Marx PA, Veazey RS: Cyclic changes in the vaginal epithelium of normal rhesus macaques. *J Endocrinol* 2006; **190**:829–35.
37 Pot B, Hertel C, Ludwig W, Descheemaeker P, Kersters K, Schleifer KH: Identification and classification of *Lactobacillus acidophilus*, *L. gasseri* and *L. johnsonii* strains by SDS-PAGE and rRNA-targeted oligonucleotide probe hybridization. *J Gen Microbiol* 1993; **139**:513–7.
38 Rush CM, Hafner LM, Timms P: Genetic modification of a vaginal strain of *Lactobacillus fermentum* and its maintenance within the reproductive tract after intravaginal administration. *J Med Microbiol* 1994; **41**:272–8.
39 Scorpio DG, Ruben DS, Liao Z, Hildreth JE, Fletcher CA: Cervicovaginal evaluation in macaques used as a model for topical microbicide safety studies. *J Med Primatol* 2008; **37**(Suppl. 1):65–73.
40 Sha BE, Zariffard MR, Wang QJ, Chen HY, Bremer J, Cohen MH, Spear GT: Female genital-tract HIV load correlates inversely with *Lactobacillus* species but positively with bacterial vaginosis and *Mycoplasma hominis*. *J Infect Dis* 2005; **191**:25–32.

41 Sobel JD, Chaim W: Vaginal microbiology of women with acute recurrent vulvovaginal candidiasis. *J Clin Microbiol* 1996; **34**:2497–9.
42 Song YL, Kato N, Matsumiya Y, Liu CX, Kato H, Watanabe K: Identification of and hydrogen peroxide production by fecal and vaginal lactobacilli isolated from Japanese women and newborn infants. *J Clin Microbiol* 1999; **37**:3062–4.
43 Taha TE, Hoover DR, Dallabetta GA, Kumwenda NI, Mtimavalye LA, Yang LP, Liomba GN, Broadhead RL, Chiphangwi JD, Miotti PG: Bacterial vaginosis and disturbances of vaginal flora: association with increased acquisition of HIV. *AIDS* 1998; **12**:1699–706.
44 Tien D, Schnaare RL, Kang F, Cohl G, McCormick TJ, Moench TR, Doncel G, Watson K, Buckheit RW, Lewis MG, Schwartz J, Douville K, Romano JW: *In vitro* and *in vivo* characterization of a potential universal placebo designed for use in vaginal microbicide clinical trials. *AIDS Res Hum Retroviruses* 2005; **21**:845–53.
45 Tsai CC, Emau P, Jiang Y, Agy MB, Shattock RJ, Schmidt A, Morton WR, Gustafson KR, Boyd MR: Cyanovirin-N inhibits AIDS virus infections in vaginal transmission models. *AIDS Res Hum Retroviruses* 2004; **20**:11–8.
46 Vasquez A, Jakobsson T, Ahrne S, Forsum U, Molin G: Vaginal *Lactobacillus* flora of healthy Swedish women. *J Clin Microbiol* 2002; **40**:2746–9.
47 Veazey RS, Klasse PJ, Ketas TJ, Reeves JD, Piatak M Jr, Kunstman K, Kuhmann SE, Marx PA, Lifson JD, Dufour J, Mefford M, Pandrea I, Wolinsky SM, Doms RW, DeMartino JA, Siciliano SJ, Lyons K, Springer MS, Moore JP: Use of a small molecule CCR5 inhibitor in macaques to treat simian immunodeficiency virus infection or prevent simian-human immunodeficiency virus infection. *J Exp Med* 2003; **198**:1551–62.
48 Veazey RS, Klasse PJ, Schader SM, Hu Q, Ketas TJ, Lu M, Marx PA, Dufour J, Colonno RJ, Shattock RJ, Springer MS, Moore JP: Protection of macaques from vaginal SHIV challenge by vaginally delivered inhibitors of virus-cell fusion. *Nature* 2005; **438**:99–102.
49 Veazey RS, Shattock RJ, Pope M, Kirijan JC, Jones J, Hu Q, Ketas T, Marx PA, Klasse PJ, Burton DR, Moore JP: Prevention of virus transmission to macaque monkeys by a vaginally applied monoclonal antibody to HIV-1 gp120. *Nat Med* 2003; **9**:343–6.
50 Zhou X, Bent SJ, Schneider MG, Davis CC, Islam MR, Forney LJ: Characterization of vaginal microbial communities in adult healthy women using cultivation-independent methods. *Microbiology* 2004; **150**:2565–73.
