# Supplementary material for: Robust Vaginal Colonization of Macaques with a Novel Vaginally Disintegrating Tablet Containing a Live Biotherapeutic Product to Prevent HIV Infection in Women
Source: PLoS One. 2015 Apr 14;10(4):e0122730. doi: 10.1371/journal.pone.0122730 (PMC4397015; doi:10.1371/journal.pone.0122730)
Supplement: S3 File — http://dx.doi.org/10.1371/journal.pone.0078817 (PDF) [file pone.0122730.s003.pdf]

PLOS | ONE

In Vivo Evaluation of Safety and Toxicity of a *Lactobacillus jensenii* Producing Modified Cyanovirin-N in a Rhesus Macaque Vaginal Challenge Model

Beda Brichacek, Laurel A. Lagenaur , Peter P. Lee, David Venzon, Dean H. Hamer

Published: November 12, 2013 • DOI: 10.1371/journal.pone.0078817

Abstract

Sexual transmission of human immunodeficiency virus type 1 (HIV-1) across the cervicovaginal mucosa in women is influenced by many factors including the microbiota and the presence of underlying inflammation. It is important that potential HIV preventative agents do not alter the mucosal environment in a way that enhances HIV acquisition. We examined the impact of a "live" microbicide on the vaginal mucosal environment in a rhesus macaque repeated vaginal simian-HIV ($SHIV_{SF162P3}$) challenge model. The microbicide contained a human vaginal *Lactobacillus jensenii* expressing the HIV-1 entry inhibitor, modified Cyanovirin-N (mCV-N), and henceforth called LB-mCV-N. Macaques were colonized vaginally each week with LB-mCV-N and sampled six days after colonization for culturable bacteria, pH and cervical-vaginal cytokines during the duration of the six-week study. We show that macaques that retained the engineered LB-mCV-N strain in their vaginal microbiota, during SHIV challenge, had lower pH, when colonization levels were higher, and had no evidence of inflammatory cytokines. Indeed, Interleukin-13, a mediator of inflammation, was detected less often in LB-mCV-N colonized macaques than in controls and we found higher levels of Interleukin 1 receptor antagonist (IL-1RA) in LB-mCV-N colonized macaques during the SHIV challenge period. We noted an inverse correlation between levels of mucosal IL-1RA and peak plasma viral load, thus higher IL-1RA correlated with lower viral load in LB-mCV-N treated macaques. These data support the use of LB-mCV-N as a safe "live" microbicide and suggest that lactobacilli themselves may positively impact the mucosal environment.

Figures

Citation: Brichacek B, Lagenaur LA, Lee PP, Venzon D, Hamer DH (2013) *In Vivo* Evaluation of Safety and Toxicity of a *Lactobacillus jensenii* Producing Modified Cyanovirin-N in a Rhesus Macaque Vaginal Challenge Model. PLoS ONE 8(11): e78817. doi:10.1371/journal.pone.0078817

Editor: Stefan Bereswill, Charité-University Medicine Berlin, Germany

Received: June 12, 2013; **Accepted:** September 16, 2013; **Published:** November 12, 2013

This is an open-access article, free of all copyright, and may be freely reproduced, distributed, transmitted, modified, built upon, or otherwise used by anyone for any lawful purpose. The work is made available under the Creative Commons CC0 public domain dedication.

Funding: NIH's Intramural AIDS Targeted Antiviral Program (IATAP) provided funding for the challenge study. This work was also supported by NIH grants 5R33AI071978-05 and 3R33AI071978-04S1. Funders had no role in study design, data collection, analysis, decision to publish or preparation of this manuscript.

Competing interests: LAL, and PPL work for Osel Inc., a company developing genetically modified vaginal Lactobacillus. BB, DV and DHH have no conflict of interest. There are no further patents, products in development or marketed products to declare. This does not alter the authors' adherence to all the PLOS ONE policies on sharing data and materials, as detailed online in the guide for authors.

Introduction

The majority of human immunodeficiency virus type 1 (HIV-1) transmissions in women occur during heterosexual intercourse when the virus penetrates mucosal surfaces of the vagina and cervix to reach target cells [1]–[4]. HIV-1 acquisition is potentiated by genital tract inflammation, which is often the result of an asymptomatic sexually transmitted infection [5]–[9]. Likewise, changes in the vaginal microbiota, found in conditions such as bacterial vaginosis, characterized by increased vaginal pH and higher levels of pro-inflammatory cytokines in cervical-vaginal secretions, lead to a higher probability of HIV-1 transmission [9]–[15]. The successful CAPRISA-004 trial, which tested 1% tenofovir gel, found that genital inflammation undermined gel effectiveness; thus, women with sufficient drug levels but inflamed epithelium were less protected than women without inflammation [16]. Finally, inflammation at the time of HIV-1 infection was found to be predictive of higher plasma viral load set point in women [17].

Early prevention trials of non-specific microbicides, such as Nonoxynol-9 (N-9) and cellulose sulfate (CS), may have contributed to increased rates of infections among users due to inflammation and disruption of the innate mucosal defenses [18]–[20]. Recently, it was also shown that N-9 and CS alter the vaginal microbiota [21]. Several groups have described methods for evaluation of biomarkers associated with cervical/vaginal mucosal barrier function and inflammation [22]–[28]. Evaluation of these biomarkers in the presence of a microbicide can be used as a guide to screen potential candidates for safety prior to their introduction into clinical settings.

We previously examined the efficacy of a live microbicide, *Lactobacillus jensenii* 1153–1666 expressing the HIV-1 entry inhibitor, modified Cyanovirin-N, hence called

LB-mCV-N, in a repeated $SHIV_{SF162P3}$ challenge vaginal model, and demonstrated that colonized animals showed a 63% reduction in acquisition of the virus and 6-fold reduced peak viral load [29], see File S1. During the SHIV challenge experiment, we examined the impact of this live microbicide, LB-mCV-N, on the vaginal microbiota, vaginal pH and vaginal biomarkers present in rhesus macaques. In this paper we present the findings of these studies.

Results

Analysis of the Culturable Vaginal Microbiome during Challenge

The vaginal microbiome is known to play an important role in the health of the lower genital tract**.** We analyzed the culturable vaginal microbiota of the experimental macaques, both those repeatedly colonized (n = 12) with LB-mCV-N and uncolonized (HEC treated) controls (n = 8) during the SHIV challenge experiment. We present the microbiota, as stacked bar graphs that represent the proportions of each bacterium and include the colony forming units (CFU) cultured from each swab (by a semi-quantitative method). Pre-challenge graphs are presented for all macaques and then subsequent microbiota each week until the macaque became infected. Animals were divided arbitrarily into those that became infected early, 1–2 challenges (Figure 1A), intermediate, 3–4 challenges (Figure 1B), late 5–6 challenges (Figure 1C) or those that remained uninfected (Figure 1D). Five control macaques and one LB-mCV-N macaque became infected early (Figure 1A). Menses is noted, when it occurred on the day of sampling (overlaying the stacked bar) or as part of daily routine observations (between bars). LB-mCV-N colonization was associated with longer time to acquisition of SHIV. Seven of eight controls were infected at early or intermediate challenges, while one control resisted infection. Conversely only three of 12 LB-mCV-N colonized macaques were infected at early or intermediate challenges, five were infected late and four resisted infection. *Lactobacillus, Streptococcus* and *Enterococcus* species, all lactic acid producing organisms were cultured from the macaque vagina. *Enterobacteriaceae,* hemolytic *Staphylococcus aureus/intermedius* and facultative anaerobic sp., including *Bacteroides sp*., *Peptostreptococcus sp*., and *Peptoniphilus sp.* were also isolated. Non-hemolytic *Staphylococcus*, *Micrococcus* and *Corynebacterium sp.* were occasionally isolated. The vaginal microbiome in the macaque is dynamic. A Shannon Diversity Index, a measure a total number of species in the community, was performed on the microbiology sample analysis. No differences in bacterial diversity were noted in the *Lactobacillus* treated vs. HEC placebo treated macaques (statistical significance p = 0.11 by the Mann-Whitney-Wilcoxon test). Thus addition of LB-mCV-N did not change the overall diversity of the vaginal microbiota.

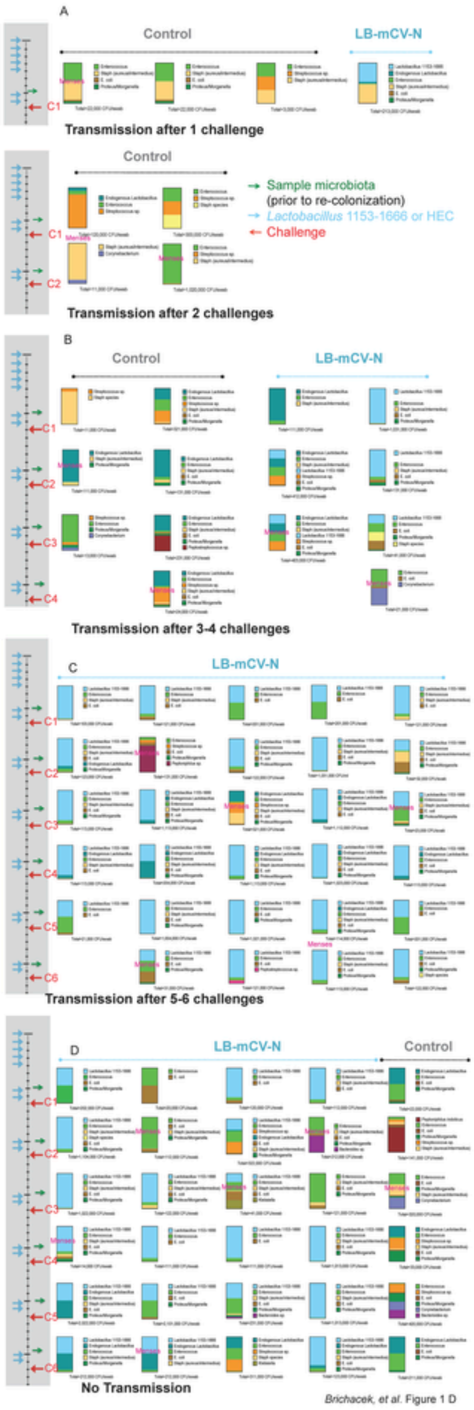

Figure 1. Culturable vaginal microbiota up to the time of transmission for mLB-CV-N colonized and control macaques.

Each vertical column represents one macaque. Samples were taken two days prior to each challenge and cultured on standard and selective plating media. Bacteria were identified by Gram stain, API biochemical analysis and 16S rRNA sequence. Stacked bar graphs are used to show the relative percentage of the culturable vaginal microbiota within the macaque at the time of sampling and the number of colony forming units (CFU) isolated per swab, prior to re-colonization. The time frame for the six $SHIV_{SF162P3}$ challenges (C1–6) is shown on the left; each challenge is represented as a red arrow, sampling is represented as a green arrow, re-colonization with LB-mCV-N or HEC treatment of macaques is represented as a light blue arrow. The first row of stacked bar graphs is pre-challenge. Macaques were arbitrarily divided into four groups, A) macaques that became infected at 1 or 2 challenges included five controls and one LB-mCV-N treated animal, B) macaques that became infected at 3–4 challenges included two controls and two LB-mCV-N treated, C) five macaques that became infected at 5–6 challenges were all treated with LB-mCV-N and D) macaques that resisted 6 challenges included four LB-mCV-N and one control. Menses that occurred on the day of sampling, is noted on the stacked bar graphs, menses that occurred during routine monitoring is noted between the stacked bar graphs. A Shannon Diversity Index was performed; no differences in bacterial diversity were noted in LB-mCV-N treated vs. the controls (p = 0.11 by Mann–Whitney-Wilcoxon test).

doi:10.1371/journal.pone.0078817.g001

Examination of the Menstrual Cycle and Influences on Microbiome and SHIV Susceptibility

Menstruation may have an effect on the stability of the vaginal microbiome, including decreasing the numbers of *Lactobacillus* and other species, as we previously have reported [30]. We observed menses during our challenge experiment and found no correlation between menstruation and SHIV infection. Notably, menstruation on the day of microbiology sampling did appear to impact the stability of the vagina microbiota in macaques that were colonized with LB-mCV-N (Figure 1, menses is noted), with a shift toward facultative anaerobic species, *Staphylococcus* sp., *Enterococcus* sp. or *Enterobacteriaceae*. Late luteal phase, which occurs just prior to menstruation was reported to be associated with increased susceptibility to SHIV vaginal infection in pigtail macaques [31]. We found that among the LB-mCV-N colonized macaques with regular menstruation cycles, 19 challenges during the luteal phase resulted in 3 infections while 8 challenges during the follicular phase produced 1 infection. This difference was not statistically significant.

Analysis of Vaginal pH Related to Levels of *Lactobacillus* Colonization

Lactobacilli are known to lower vaginal pH in humans. To determine whether LB-mCV-N played a role in lowering vaginal pH in the macaque model, we first measured vaginal pH in macaques that were originally colonized with endogenous strains of *Lactobacillus* and then recolonized with LB-mCV-N strain and showed that both types of lactobacilli contribute similarly to vaginal pH (Figure 2A). Vaginal pH in macaques that were colonized with the highest levels of LB-mCV-N, at least 10^6 colony forming units (CFU), were lower pH by an estimated 0.4 pH units from those with no colonization (p = 0.0058, Figure 2B). The trend across all the LB-mCV-N levels between no colonization and having at least 10^6 CFU was neither significantly positive nor negative.

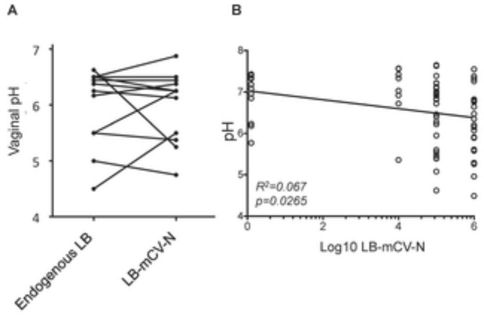

Figure 2. Influences of Lactobacillus on the vaginal pH of macaques.
A) Vaginal pH was the same for macaques colonized with LB-mCVN or endogenous LB. Macaques colonized with endogenous *Lactobacillus* (LB) followed by colonization with LB-mCV-N had similar pH, no statistical different was noted in the vaginal pH with the different *Lactobacillus* species. B) Vaginal pH was measured and plotted against the levels of colonization with LB-mCV-N. Values on the far left represent times when macaques had no LB-mCV-N, and the other values are log levels of colony forming units (CFUs) of LB-mCV-N. Lower pH trended with LB-mCV-N. By simple linear regression, lower pH trended with LB-mCV-N (R^2 = 0.067, slope p = 0.027). Macaques that were colonized with the highest levels of LB-mCV-N, at least 10^6 colony forming units (CFU), were lower by an estimated 0.4 pH units from those with no colonization (p = 0.0058 by repeated measures analysis of variance).
doi:10.1371/journal.pone.0078817.g002

Examination of Mucosal Biomarkers of Inflammation during Colonization and SHIV Challenge

Increases in the levels of proinflammatory or anti-inflammatory cytokines in the cervical-vaginal environment can enhance or reduce the susceptibility to HIV. To determine whether the LB-mCV-N microbicide altered biomarkers in the mucosal environment, we measured cytokines known for their effect on HIV transmission. We used a Luminex 23-plex assay to measure G-CSF, GM-CSF, IFN-γ, IL-1β, IL-1RA, IL-2, IL-4, IL-5, IL-6, IL-8, IL-10, IL12/23(p-40), IL-13, IL-15, IL-17, MCP-1, MIP-1β, MIP-1α, sCD40L, TGFα, TNFα, VEGF, and IL-18, in the CVLs of control and LB-mCV-N colonized macaques upon initial colonization and during the challenge experiment. Biomarkers were analyzed in an exploratory mode without correction for multiple comparisons but in consideration of independent results from previous studies.

During the period of initial colonization, we observed that IL-13, a mediator of allergic inflammation, was detectable in the CVL of 7/8 control macaques, but was only detectable in 3/12 CVLs from LB-mCV-N colonized macaques (p = 0.0198* Fishers t-test; data not shown). Thus it appears that colonization with LB-mCV-N suppressed the levels of the proinflammatory IL-13. No other cytokines were altered upon initial colonization (pre-challenge values).

During the time period of the repeated vaginal SHIV challenge, we measured cytokines in the CVL's of control and LB-mCV-N colonized macaques. We subtracted the pre-challenge cytokine background and looked for changes that occurred due to the interaction of the live microbicide on the epithelium in the presence of SHIV virus. In macaques that were dosed repeatedly with LB-mCV-N and challenged with SHIV, IL-1RA was found to be upregulated in CVLs compared to the levels in CVLs of controls (p = 0.013) (Figure 3). There were no statistical changes noted in any other proinflammatory biomarkers IL-1β, IL-6, TNFα, GM-CSF, IFN-γ, IL-8, IL12/23(p-40), IL-17, and IL-18, measured prior to and during the repeated challenges. Table 1 shows the minimum and maximum ranges and median levels of proinflammatory cytokines in the macaques. The table is divided to show the proinflammatory cytokine levels (A) prior to SHIV challenge in all animals and (B) during the challenges before transmission. Note that three control macaques and one LB-mCV-N macaque became infected at the first challenge and thus were excluded from this analysis.

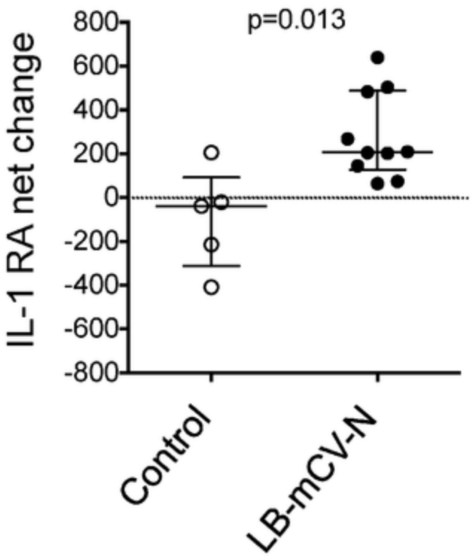

Figure 3. IL-1RA concentrations from cervical vaginal lavage (CVL) samples of control or LB-mCV-N colonized macaques during the SHIV challenge experiment.

IL-1RA levels are reported as the net change in cytokine with the baseline (pre-challenge) values subtracted. IL-1RA was significantly higher in LB-mCV-N colonized macaques during challenge, p = 0.013. Note: SHIV transmission occurred at the first challenge in four animals (three controls and one LB-mCV-N), thus there were insufficient values available for baseline subtraction, and these animals were excluded from the analysis. One LB-mCV-N animal was missing a pre-challenge sample and was also excluded.

doi:10.1371/journal.pone.0078817.g003

	IL-1β	IL-6	TNFα	IL-13	GM-CSF	IFNγ	IL-8	IL-12 p40	IL-17	IL-18
**A. Colonized and control animals, prior to SHIV challenges**										
**LB-CV-N colonized animals**										
min	<0.07	<0.03	<0.5	<0.01	<0.03	<0.06	111	0.34	<0.02	1.0
max	243.9	917.8	25.8	2.0	8.5	1.8	10706	13.5	0.6	499
median	0.7	3.5	0.3	0.0	0.4	0.3	360	2.0	0.0	10.1
n	12	12	12	12	12	12	12	12	12	12
**Non-colonized control animals**										
min	<0.07	0.56	<0.5	<0.01	<0.03	<0.06	43.8	0.3	<0.02	0.9
max	148.8	48.6	11.3	1.5	2.9	1.0	2572	61.3	1.4	173
median	1.1	8.7	2.1	0.2	0.3	0.2	298	2.6	0.1	15.0
n	8	8	8	8	8	8	8	8	8	8
**B. Colonized and control animals during SHIV challenges, preinfection**										
**LB-CV-N colonized animals**										
	IL-1β	IL-6	TNFα	IL-13	GM-CSF	IFNγ	IL-8	IL-12 p40	IL-17	IL-18
min	0.8	2.1	<0.5	<0.01	<0.03	<0.06	489	0.9	<0.02	2.9
max	140	587	32.6	0.7	3.5	0.8	6368	7.0	0.3	104
median	2.9	36.9	0.6	0.0	0.6	0.2	1187	2.3	0.1	28.9
n	11	11	11	11	11	11	11	11	11	11
**Non-colonized control animals**										
min	1.6	3.7	<0.5	<0.01	<0.03	<0.06	160.0	1.0	<0.02	0.6
max	23.4	66.8	7.1	1.8	3.2	1.7	1235	7.5	0.6	138
median	3.0	8.6	0.4	0.4	0.1	0.4	347	1.9	0.1	11.9
n	5	5	5	5	5	5	5	5	5	5

doi:10.1371/journal.pone.0078817.t001

Table 1. Pro-inflammatory cytokine levels in CVL (pg/ml).

doi:10.1371/journal.pone.0078817.t001

During our challenge experiment, we reported that LB-mCV-N colonized macaques required more SHIV challenges to become infected (as shown in Figure 1) and LB-mCV-N colonized macaques with breakthrough infection had 6-fold lower viral load than control macaques [29]. We asked whether the elevated mucosal IL-1RA levels correlated with time to transmission or reduction in peak viral load. We found no correlation between IL-1RA levels and overall time to transmission. Interestingly mucosal IL-1RA levels in the CVL's of macaques colonized with LB-mCV-N were inversely correlated with peak plasma viral load (Figure 4A). This correlation was not seen in the CVLs of control macaques (Figure 4B). Thus, during SHIV challenge, elevation in IL1-RA in CVL, associated with LB-mCV-N colonization, was also associated with lower peak viral load (R squared = 0.5766, p = 0.0289) in macaques with breakthrough infection.

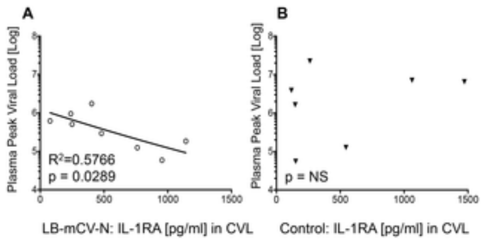

Figure 4. Higher mucosal IL-1RA was predictive of lower peak viral load.
Macaques that were colonized with LB-mCV-N and experienced breakthrough infection had 6-fold lower viral loads than control macaques [29]. Mucosal IL-1RA was measured during SHIV challenge prior to infection and the average value was plotted against the peak viral load. We found that higher levels of mucosal IL-1RA prior to infection were correlated with a lower peak viral load. A) Levels of mucosal IL-1RA [pg/ml], found in CVLs of macaques colonized with LB-mCV-N, were inversely correlated with peak plasma viral load, thus higher IL-1RA prior to infection correlated with lower viral load B) No correlation with peak plasma viral load was found in mucosal IL-1RA, found in CVL of uncolonized control macaques.
doi:10.1371/journal.pone.0078817.g004

Discussion

One currently promising approach to the prevention of HIV transmission is the use of topical microbicides which, when applied on mucosal surfaces, block or inactivate the virus before it can reach a target cell. Irrespective of their mechanism and efficacy of viral inhibition, it is essential that such microbicides not alter the healthy microbiota or harm the mucosal environment in a way that could potentiate infection. The importance of careful safety testing in suitable animal models is highlighted by the unfortunate experience with two previously examined microbicides, N-9 and CS, both of which may have enhanced HIV acquisition in human clinical trials. Subsequent studies showed that these agents were associated with increased concentrations of pro-inflammatory cytokines in vaginal fluids [20], [23], [24] and may also have altered the vaginal microbiota, with decreases in naturally protective *Lactobacillus* [21]. The first successful human microbicide trial, CAPRISA-004, used 1% tenofovir gel, which does not have such harmful effects; however, the modest efficacy that was observed in this trial may be in part attributable to existing cervical-vaginal inflammation unrelated to the gel [16]. Thus, finding ways to reduce cervical-vaginal inflammation may critical to a comprehensive HIV prevention program.

We have recently described a successful test of a live microbicide using *Lactobacillus* as a delivery system for the HIV-1 entry inhibitor cyanovirin-N (LB-mCV-N), in a macaque model [29]. The purpose of this study was to determine the impact of vaginal colonization with the engineered LB-mCV-N on the vaginal environment, specifically on the vaginal microbiota, vaginal pH, and the extracellular components of innate immunity to determine safety.

Although we were able to achieve high numbers of LB-mCV-N in the macaque vaginal environment, the LB-mCV-N did not alter the overall species diversity or lower pH to the levels seen in women. Mirmonsef *et al*. reported that rhesus macaques (and pigtail macaques) have much lower levels of vaginal glycogen and lower lactic acid levels than humans [32]. Because glycogen serves as a carbon source for the production of lactic acid, the LB-mCV-N although capable of making high levels of lactic acid, [33] did not in this model, thus no concomitant (or only a modest) drop in pH occurred. In our study, cultures from the vaginas of control macaques contained mainly *Streptococcus, Staphylococcus,* and *Enterococcus species*, while three out of eight had endogenous *Lactobacillus sp*., thus the vaginal microbial composition of our experimental animals corresponds well to the previous reports [30], [32], [34]–[36].

Our live microbicide produces mCV-N constitutively on the cervical-vaginal mucosa and we found no evidence of local inflammation in this repeated challenge model [29]. A previous report suggested that CV-N (produced in *E. coli*) may not be safe for use as a topical microbicide, because when cultured with peripheral blood mononuclear cells (PBMCs) in an *in vitro* culture system the native CV-N protein activated the expression of multiple cytokines (IL-1β, IL-1RA, IL-2, IL-4, IL-5, IL-7, IL-8, IL-9, IL-10, IL-12, IL-14, IL-15, IL-17, Eotaxin, G-CSF, GM-CSF, IFN-γ, IP-10, MCP-1, MIP-1β, MIP-1α, PDGF, RANTES, TNFα, and VEGF [37]. In contrast to the *in vitro* PBMC culture system we found that *in vivo*, there was no evidence of immune activation by the mCV-N produced *in situ* by the LB-mCV-N bacteria. Indeed, the most noteworthy comparison was an apparent decrease in IL-13 ($p = 0.0198$*), in the CVLs of LB-mCV-N colonized macaques. In addition, with repeated dosing of LB-mCV-N during the challenge experiment we observed an increase in the levels of an anti-inflammatory cytokine IL-1RA ($p = 0.013$) over background, that was not observed with sequential measurements of cytokines in our first study [29]. We did not find significant elevation of any other cytokines that were measured. Thus, the PBMC *in vitro* system [37] was not predictive of what occurs *in vivo* on the mucosa, and may instead reflect endotoxin contamination present in the native CV-N sample produced in *E. coli*.

A potentially more useful system for testing microbicides was described by Fichorova *et al*. and involved the generation and characterization of several immortalized epithelial cell lines from human vagina, ectocervix and endocervix [38], [39]. These cell lines may be used to measure cytokines for the safety evaluation of topical microbicides, including live biotherapeutic products such as LB-mCV-N (*L. jensenii* 1153–1666) [40]. Yamamoto *et al*. reported that both LB-mCV-N, and the native/parental strain *L. jensenii* 1153 did not induce proinflammatory proteins, such as IL-8, TNF-α, and IL-6 [40]. Both *L. jensenii* 1153 and LB-mCV-N produced constant levels of IL-1RA when cultured with vaginal (Vk2/E6/E7) epithelium cells [40]. IL-1RA is believed to counteract the inflammatory effects of IL-1 proteins [41] and here we show an inverse correlation between median vaginal IL-1RA levels and subsequent peak viral load. This finding is consistent with results of *in vitro* experiments, which showed that IL-1RA blocked IL-1-mediated inductive effects on HIV virus production [42]. We speculate that the increased IL-1RA present in the mucosa at the time of SHIV infection in LB-mCV-N colonized macaques could have reduced inflammation leading to a smaller initial nidus of infection and subsequent modest reduction in peak viral load.

Our finding of decreased IL-13 is consistent with the increasing body of literature on *Lactobacillus* probiotic strains and thus is likely not associated with mCV-N protein produced *in situ* by LB-mCV-N, but instead by the *Lactobacillus* themselves. Decreases in IL-13 production was described as a property of various *Lactobacillus* strains, mainly in the connection with the suppression of allergic reactions and shift from TH2 towards TH1 type responses to a more balanced cytokine profile [43]–[47].

In these *in vivo* experiments mCV-N appeared to be safe, and did not trigger any proinflammatory changes to the local mucosal environment. A live microbicide approach that uses vaginal *Lactobacillus* as a delivery platform for proteins that inhibit HIV entry, may work in multiple ways; live microbicides may improve vaginal health in humans by reconstituting the vaginal microbiota, lowering pH and inflammation and delivering the anti-viral protein *in situ* at the site of HIV entry.

Methods

Ethics Statement

The twenty female Chinese origin rhesus macaques (Macaca mulatta), used in this study were housed at BIOQUAL, Inc., in accordance with the recommendations of

the Association for Assessment and Accreditation of Laboratory Animal Care International Standards and with the recommendations in the Guide for the Care and Use of Laboratory Animals of the United States – National Institutes of Health. The Institutional Animal Use and Care Committee of BIOQUAL approved these experiments (study #3516). When immobilization was necessary, the animals were sedated by intramuscularly injection with 10 mg/kg of ketamine HCl and 1 mg/kg acepromazine. All efforts were made to minimize suffering. Details of animal welfare and steps taken to ameliorate suffering were in accordance with the recommendations of the Weatherall report, "The use of non-human primates in research". Animals were housed in an air-conditioned facility with an ambient temperature of 21–25°C, a relative humidity of 40%–60% and a 12 h light/dark cycle. Animals were socially housed when possible or individually housed if no compatible pairing could be found. The animals were housed in suspended stainless steel wire-bottomed cages and provided with a commercial primate diet and fresh fruit twice daily, with water freely available at all times.All NHPs housed at BIOQUAL, Inc. receive bi-monthly behavioral assessments from one of two fulltime behaviorists. All NHPs receive at least three commercially available pet toys in their cages at all times to manipulate as part of their enriched environment. Choosing from a wide array of toys, each animal is provided one hard toy (i.e. hard plastic), one soft toy (i.e. soft rubber), and a third toy (i.e. hard plastic or soft rubber) chosen by the caretaker assigned to each animal. NHPs are given destructible enrichment as a way for them to alter their environment, release aggression/tension, and forage through. The destructible enrichment provided includes items such as cardboard, shredded paper, and phonebooks. Soft toys, such as fleece blankets and soft pet toys, are available for comfort to all NHPs. Many foraging opportunities are made available to all NHPs. Treats used for foraging and in feeders include, but are not limited to: Bunny Blocks, Nutra Blocks, Crumble Disks, PRIMA Treats, Fruity Gems, Fleece Foraging Crumbles, wax moth larvae (all available through BIOSERV), raisins, popcorn, nuts, birdseed, oatmeal, hardboiled eggs, species-specific food items, etc. All animals observed to exhibit any abnormal behavior(s) deemed detrimental to the physical and/or mental health of the individual are prescribed specific enrichment by the behavioral staff in order to decrease/eliminate/redirect such behaviors.

Following the final SHIV challenge, macaques were held for four additional weeks to monitor viral infection and viral set point. The viral set point was designed as the time point for euthanasia to avoid pain or suffering associated with SHIV clinical sequelae. Euthanasia was performed by IV injection of a lethal dose of pentobartbital (Beuthanasia-D) and cardiac arrest was confirmed by auscultation (in agreement with the 2007 AVMA Guidelines for Euthanasia).

Study Animals

The colonization and $SHIV_{SF162P3}$ challenge study has been previously described [29], four control macaques from the original study were not included in this analysis because insufficient pre-treatment samples were available. Swab samples for microbiological determination were collected first, followed by cervical-vaginal lavage (CVL), typically on a Monday. Animals were re-colonized with LB-mCV-N delivered in 3% hydroxyethylcellulose (HEC) gel for two days after sample collection, (Monday and Tuesday), SHIV challenge occurred on Wednesday at least 24 hrs after re-colonization. These additional doses were intended to standardize the levels of LB-mCV-N among animals, and also to replenish the LB-mCV-N that was removed during CVL and swab sampling. Except for week one, the culturable microbiota shown in Figure 1, represents a sample taken six days after colonization. Control macaques received only 3% HEC gel on the same schedule as the experimental group.

Microbiology

The vaginal microbiota of each animal was sampled each week using the Port-A-Cul™ anaerobic collection and transport system (Becton Dickinson, Cockeysville, MD, USA). Swabs were collected and transported within 8 hrs. The swabs were plated using a four quadrant plating technique for isolation of aerobic bacteria on tryptic soy agar with 5% sheep blood agar, phenylethyl alcohol agar (PEA) with 5% sheep blood, MacConkey agar, and MRS agar (Becton Dickinson, Franklin Lakes, NJ). For isolation of anaerobic organisms the samples were plated to Brucella blood agar, PEA agar with 5% sheep blood, laked blood with kanamycin and vancomycin agar, and bacteroides bile esculin agar (Anaerobe Systems Morgan Hill, CA). Cultures on the primary plates were screened, and sub-cultured for isolation of pure cultures. Gram stain and API bacterial identification test strips (Biomerieux, St Louis, MO) were used for preliminary identification of the bacterial isolates. Aero-tolerance determination was used for primary facultative anaerobic bacterial identification, followed by Gram stain and API 20A identification kit. Bacterial identification to the species level was performed using 16S rRNA gene sequencing as previously described [30].

Semi -quantitative measurements of the microbiota were made on the growth in each quadrant of the plate, based on standards containing serial dilutions of *Lactobacillus jensenii* 1153–1666 plated with the same quadrant plating technique. Growth in quadrant 4 represented at least 10^6 CFU, quadrant 3, 10^5 CFU, quadrant 2, 10^4 CFU, and quadrant 1, 10^3 CFU. Stacked bar graphs are used to represent the proportional numbers of organisms found in each culture and semi-quantitative counts. Each species identified is labeled and color-coded in Figure 1. A Shannon diversity index was performed using SAS Statistical software.

Vaginal pH

Vaginal pH was monitored using a portable HI 99181 Skin pH Meter Hanna Instrument® (Woonsocket, RI).

Menstruation Evaluation

Menses was monitored by inserting a clean saline dampened cotton-tipped applicator into the monkey's vagina. The swab was then removed and a recording from 0 to 3 was made (0 = no blood, 1 = slight, 2 = moderate, 3 = heavy bleeding). The monkeys were trained for this procedure by giving food as a reward. Late luteal phase (occurring prior to menses) has been reported to be associated with increased risk of vaginal SHIV infection [31].

Cytokine and immunoglobulin measurements from Cervical Vaginal Lavage (CVL).

CVLs were taken by instilling 2 mL of PBS spiked with complete, EDTA-free protease Inhibitor (Roche Applied Science, Indianapolis, IN) into the vagina, flushing it back and forth four times, and then collecting the fluids on ice. Samples were clarified by centrifugation at 820×g at +4°C and snap frozen on dry ice, prior to storage at −80°C until analysis. The levels of 23 cytokines in CVLs were determined from 20 of the macaques used in the challenge experiment, with a multiplexed fluorescent microsphere immunoassay using the Luminex 100 system (Luminex Corporation, Austin, TX) using Milliplex™ MAP Non-human primate cytokine kit (Millipore). Biochemical markers and cytokines that were measured include Granulocyte Colony Stimulating Factor (G-CSF), Granulocyte-Macrophage Colony Stimulating Factor GM-CSF, Interferon gamma (IFN-γ), Interleukin 1 beta (IL-1β), Interleukin 1 Receptor Antagonist (IL-1RA), Interleukin 2 (IL-2), Interleukin 4 (IL-4*), Interleukin 5 (IL-5), Interleukin 6 (IL-6), Interleukin 8 (IL-8), Interleukin 10 (IL-10), Interleukin 12/23 (IL12/23(p-40), Interleukin 13 (IL-13), Interleukin 15 (IL-15), Interleukin 17 (IL-17), Monocyte Chemoattractant Protein -1 (MCP-1), Macrophage Inflammatory Protein 1 beta (MIP-1β), Macrophage Inflammatory Protein 1 alpha (MIP-1α), soluble CD 40 Cluster of Differentiation Ligand (sCD40L), Transforming Growth Factor alpha (TGFα), Tumor Necrosis Factor alpha (TNFα), Vascular Endothelial Growth Factor

(VEGF), and Interleukin 18 (IL-18). *(IL-4 was not detected or below the level of detection). Data were collected and analyzed with Bioplex Manager v3.0 software (Bio-Rad, Hercules, CA) using a 5-parameter fitting algorithm. To account for possible differences between tests, samples were analyzed in overlapping batches testing all the time points from the individual animals at the same time. All p values of 0.05 or less have been reported, and have not been corrected for multiple comparisons. Because of multiple (23) measurements, a cytokine/biomarker that reaches a value 0.05 may not be significant if a Bonferroni correction is applied.

Statistical Analysis

The Statistics and Data Management Section of National Institutes of Heath performed statistical analyses on the data.

Supporting Information

File_S1.pdf

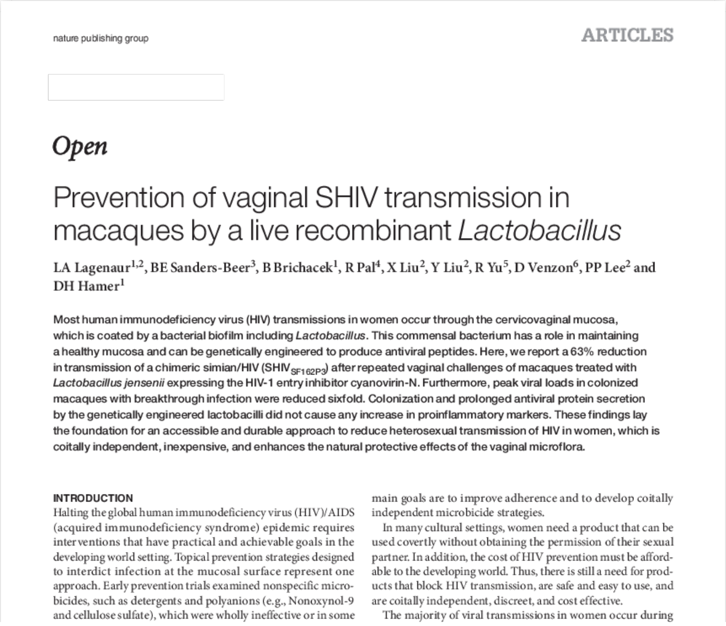

nature publishing group ARTICLES

Open

Prevention of vaginal SHIV transmission in macaques by a live recombinant *Lactobacillus*

LA Lagenaur[1,2], BE Sanders-Beer[3], B Brichacek[1], R Pal[4], X Liu[2], Y Liu[2], R Yu[5], D Venzon[6], PP Lee[2] and DH Hamer[1]

Most human immunodeficiency virus (HIV) transmissions in women occur through the cervicovaginal mucosa, which is coated by a bacterial biofilm including *Lactobacillus*. This commensal bacterium has a role in maintaining a healthy mucosa and can be genetically engineered to produce antiviral peptides. Here, we report a 63% reduction in transmission of a chimeric simian/HIV ($SHIV_{SF162P3}$) after repeated vaginal challenges of macaques treated with *Lactobacillus jensenii* expressing the HIV-1 entry inhibitor cyanovirin-N. Furthermore, peak viral loads in colonized macaques with breakthrough infection were reduced sixfold. Colonization and prolonged antiviral protein secretion by the genetically engineered lactobacilli did not cause any increase in proinflammatory markers. These findings lay the foundation for an accessible and durable approach to reduce heterosexual transmission of HIV in women, which is coitally independent, inexpensive, and enhances the natural protective effects of the vaginal microflora.

INTRODUCTION

Halting the global human immunodeficiency virus (HIV)/AIDS (acquired immunodeficiency syndrome) epidemic requires interventions that have practical and achievable goals in the developing world setting. Topical prevention strategies designed to interdict infection at the mucosal surface represent one approach. Early prevention trials examined nonspecific microbicides, such as detergents and polyanions (e.g., Nonoxynol-9 and cellulose sulfate), which were wholly ineffective or in some main goals are to improve adherence and to develop coitally independent microbicide strategies.

In many cultural settings, women need a product that can be used covertly without obtaining the permission of their sexual partner. In addition, the cost of HIV prevention must be affordable to the developing world. Thus, there is still a need for products that block HIV transmission, are safe and easy to use, and are coitally independent, discreet, and cost effective.

The majority of viral transmissions in women occur during

figshare download

Prevention of vaginal SHIV transmission in macaques by a live recombinant *Lactobacillus*.

File S1.
Prevention of vaginal SHIV transmission in macaques by a live recombinant *Lactobacillus*.
doi:10.1371/journal.pone.0078817.s001
(PDF)

Acknowledgments

We thank Laurent Pessaint at BIOQUAL for preparation of LB-mCV-N, placebo and animal blood processing, Matt Collins, and Jermaine Hoes for animal sampling and care. We also thank Brigitte Sanders-Beer for aid in the execution of this study.

Author Contributions

Performed the experiments: BB LAL. Analyzed the data: DV LAL BB. Contributed reagents/materials/analysis tools: DV. Wrote the paper: LAL BB. Conception and design of the work, acquisition of data, or analysis and interpretation of data: BB LAL. Analysis and interpretation of data: DV. Drafting the article or revising it critically for important intellectual content: DH PPL.

References

1. Gray RH, Wawer MJ, Brookmeyer R, Sewankambo NK, Serwadda D, et al. (2001) Probability of HIV-1 transmission per coital act in monogamous, heterosexual, HIV-1-discordant couples in Rakai, Uganda. Lancet 357: 1149–1153. doi: 10.1016/s0140-6736(00)04331-2.
CrossRef • PubMed/NCBI • Google Scholar
2. Hladik F, Hope TJ (2009) HIV infection of the genital mucosa in women. Curr HIV/AIDS Rep 6: 20–28. doi: 10.1007/s11904-009-0004-1.
CrossRef • PubMed/NCBI • Google Scholar
3. Abdool Karim Q, Sibeko S, Baxter C (2010) Preventing HIV infection in women: a global health imperative. Clin Infect Dis 50 Suppl 3S122–129. doi: 10.1086/651483.
CrossRef • PubMed/NCBI • Google Scholar
4. Hughes JP, Baeten JM, Lingappa JR, Magaret AS, Wald A, et al. (2012) Determinants of per-coital-act HIV-1 infectivity among African HIV-1-serodiscordant couples. J Infect Dis 205: 358–365. doi: 10.1093/infdis/jir747.
CrossRef • PubMed/NCBI • Google Scholar
5. Cohen MS (2004) HIV and sexually transmitted diseases: lethal synergy. Top HIV Med 12: 104–107.
CrossRef • PubMed/NCBI • Google Scholar
6. Galvin SR, Cohen MS (2004) The role of sexually transmitted diseases in HIV transmission. Nat Rev Microbiol 2: 33–42. doi: 10.1038/nrmicro794.
CrossRef • PubMed/NCBI • Google Scholar
7. de Jong MA, Geijtenbeek TB (2009) Human immunodeficiency virus-1 acquisition in genital mucosa: Langerhans cells as key-players. J Intern Med 265: 18–28. doi: 10.1111/j.1365-2796.2008.02046.x.
CrossRef • PubMed/NCBI • Google Scholar
8. Mayer KH, Venkatesh KK (2011) Interactions of HIV, other sexually transmitted diseases, and genital tract inflammation facilitating local pathogen transmission and acquisition. Am J Reprod Immunol 65: 308–316. doi: 10.1111/j.1600-0897.2010.00942.x.
CrossRef • PubMed/NCBI • Google Scholar
9. Thurman AR, Doncel GF (2011) Innate immunity and inflammatory response to Trichomonas vaginalis and bacterial vaginosis: relationship to HIV acquisition. Am J Reprod Immunol 65: 89–98. doi: 10.1111/j.1600-0897.2010.00902.x.
CrossRef • PubMed/NCBI • Google Scholar
10. Hawes SE, Hillier SL, Benedetti J, Stevens CE, Koutsky LA, et al. (1996) Hydrogen peroxide-producing lactobacilli and acquisition of vaginal infections. J Infect Dis 174: 1058–1063. doi: 10.1093/infdis/174.5.1058.
CrossRef • PubMed/NCBI • Google Scholar
11. Hillier SL (1998) The vaginal microbial ecosystem and resistance to HIV. AIDS Res Hum Retroviruses 14 Suppl 1S17–21.
CrossRef • PubMed/NCBI • Google Scholar
12. Taha TE, Hoover DR, Dallabetta GA, Kumwenda NI, Mtimavalye LA, et al. (1998) Bacterial vaginosis and disturbances of vaginal flora: association with increased acquisition of HIV. AIDS 12: 1699–1706. doi: 10.1097/00002030-199813000-00019.
CrossRef • PubMed/NCBI • Google Scholar
13. Martin HL, Richardson BA, Nyange PM, Lavreys L, Hillier SL, et al. (1999) Vaginal lactobacilli, microbial flora, and risk of human immunodeficiency virus type 1 and sexually transmitted disease acquisition. J Infect Dis 180: 1863–1868. doi: 10.1086/315127.
CrossRef • PubMed/NCBI • Google Scholar
14. Boskey ER, Cone RA, Whaley KJ, Moench TR (2001) Origins of vaginal acidity: high D/L lactate ratio is consistent with bacteria being the primary source. Hum Reprod 16: 1809–1813. doi: 10.1093/humrep/16.9.1809.
CrossRef • PubMed/NCBI • Google Scholar
15. Atashili J, Poole C, Ndumbe PM, Adimora AA, Smith JS (2008) Bacterial vaginosis and HIV acquisition: a meta-analysis of published studies. Aids 22: 1493–1501. doi: 10.1097/qad.0b013e3283021a37.
CrossRef • PubMed/NCBI • Google Scholar
16. Karim SS (2012) CAPRISA 004 TWO YEARS ON: WHAT WERE THE LESSONS, WHAT ARE THE IMPLICATIONS? Microbicides 2012. Sydney, Australia.
17. Roberts L, Passmore JA, Mlisana K, Williamson C, Little F, et al. (2012) Genital tract inflammation during early HIV-1 infection predicts higher plasma viral load set point in women. J Infect Dis 205: 194–203. doi: 10.1093/infdis/jir715.
CrossRef • PubMed/NCBI • Google Scholar
18. Hillier SL, Moench T, Shattock R, Black R, Reichelderfer P, et al. (2005) In vitro and in vivo: the story of nonoxynol 9. J Acquir Immune Defic Syndr 39: 1–8. doi: 10.1097/01.qai.0000159671.25950.74.
CrossRef • PubMed/NCBI • Google Scholar
19. Van Damme L, Govinden R, Mirembe FM, Guedou F, Solomon S, et al. (2008) Lack of effectiveness of cellulose sulfate gel for the prevention of vaginal HIV transmission. N Engl J Med 359: 463–472. doi: 10.1056/nejmoa0707957.
CrossRef • PubMed/NCBI • Google Scholar
20. Mesquita PM, Cheshenko N, Wilson SS, Mhatre M, Guzman E, et al. (2009) Disruption of tight junctions by cellulose sulfate facilitates HIV infection: model of microbicide safety. J Infect Dis 200: 599–608. doi: 10.1086/600867.
CrossRef • PubMed/NCBI • Google Scholar
21. Ravel J, Gajer P, Li F, Mauck CK, Koenig SS, et al.. (2012) Twice-Daily Application of HIV Microbicides Alter the Vaginal Microbio. MBio 3.
22. Fichorova RN, Tucker LD, Anderson DJ (2001) The molecular basis of nonoxynol-9-induced vaginal inflammation and its possible relevance to human immunodeficiency virus type 1 transmission. J Infect Dis 184: 418–428. doi: 10.1086/322047.
CrossRef • PubMed/NCBI • Google Scholar
23. Fichorova RN (2004) Guiding the vaginal microbicide trials with biomarkers of inflammation. J Acquir Immune Defic Syndr 37 Suppl 3S184–193. doi:

10.1097/00126334-200410013-00010.
CrossRef • PubMed/NCBI • Google Scholar

24. Fichorova RN, Bajpai M, Chandra N, Hsiu JG, Spangler M, et al. (2004) Interleukin (IL)-1, IL-6, and IL-8 predict mucosal toxicity of vaginal microbicidal contraceptives. Biol Reprod 71: 761–769. doi: 10.1095/biolreprod.104.029603.
CrossRef • PubMed/NCBI • Google Scholar
25. Trifonova RT, Bajpai M, Pasicznyk JM, Chandra N, Doncel GF, et al. (2007) Biomarkers of leukocyte traffic and activation in the vaginal mucosa. Biomarkers 12: 608–622. doi: 10.1080/13547500701600670.
CrossRef • PubMed/NCBI • Google Scholar
26. Dezzutti CS, Hendrix CW, Marrazzo JM, Pan Z, Wang L, et al. (2011) Performance of swabs, lavage, and diluents to quantify biomarkers of female genital tract soluble mucosal mediators. PLoS One 6: e23136. doi: 10.1371/journal.pone.0023136.
CrossRef • PubMed/NCBI • Google Scholar
27. Scott ME, Wilson SS, Cosentino LA, Richardson BA, Moscicki AB, et al. (2011) Interlaboratory reproducibility of female genital tract cytokine measurements by Luminex: Implications for microbicide safety studies. Cytokine 56: 430–434. doi: 10.1016/j.cyto.2011.06.011.
CrossRef • PubMed/NCBI • Google Scholar
28. Su HI, Schreiber CA, Fay C, Parry S, Elovitz MA, et al. (2011) Mucosal integrity and inflammatory markers in the female lower genital tract as potential screening tools for vaginal microbicides. Contraception 84: 525–532. doi: 10.1016/j.contraception.2011.02.010.
CrossRef • PubMed/NCBI • Google Scholar
29. Lagenaur LA, Sanders-Beer BE, Brichacek B, Pal R, Liu X, et al.. (2011) Prevention of vaginal SHIV transmission in macaques by a live recombinant Lactobacillus. Mucosal Immunol.
30. Yu RR, Cheng AT, Lagenaur LA, Huang W, Weiss DE, et al. (2009) A Chinese rhesus macaque (Macaca mulatta) model for vaginal Lactobacillus colonization and live microbicide development. J Med Primatol 38: 125–136. doi: 10.1111/j.1600-0684.2008.00316.x.
CrossRef • PubMed/NCBI • Google Scholar
31. Vishwanathan SA, Guenthner PC, Lin CY, Dobard C, Sharma S, et al. (2011) High susceptibility to repeated, low-dose, vaginal SHIV exposure late in the luteal phase of the menstrual cycle of pigtail macaques. J Acquir Immune Defic Syndr 57: 261–264. doi: 10.1097/qai.0b013e318220ebd3.
CrossRef • PubMed/NCBI • Google Scholar
32. Mirmonsef P, Gilbert D, Veazey RS, Wang J, Kendrick SR, et al.. (2011) A Comparison of Lower Genital Tract Glycogen and Lactic Acid Levels in Women and Macaques: Implications for HIV and SIV Susceptibility. AIDS Res Hum Retroviruses.
33. Liu X, Lagenaur LA, Simpson DA, Essenmacher KP, Frazier-Parker CL, et al. (2006) Engineered vaginal lactobacillus strain for mucosal delivery of the human immunodeficiency virus inhibitor cyanovirin-N. Antimicrob Agents Chemother 50: 3250–3259. doi: 10.1128/aac.00493-06.
CrossRef • PubMed/NCBI • Google Scholar
34. Doyle L, Young CL, Jang SS, Hillier SL (1991) Normal vaginal aerobic and anaerobic bacterial flora of the rhesus macaque (Macaca mulatta). J Med Primatol 20: 409–413.
CrossRef • PubMed/NCBI • Google Scholar
35. Spear GT, Gilbert D, Sikaroodi M, Doyle L, Green L, et al. (2010) Identification of rhesus macaque genital microbiota by 16S pyrosequencing shows similarities to human bacterial vaginosis: implications for use as an animal model for HIV vaginal infection. AIDS Res Hum Retroviruses 26: 193–200. doi: 10.1089/aid.2009.0166.
CrossRef • PubMed/NCBI • Google Scholar
36. Gravett MG, Jin L, Pavlova SI, Tao L (2012) Lactobacillus and Pediococcus species richness and relative abundance in the vagina of rhesus monkeys (Macaca mulatta). J Med Primatol 41: 183–190. doi: 10.1111/j.1600-0684.2012.00537.x.
CrossRef • PubMed/NCBI • Google Scholar
37. Huskens D, Vermeire K, Vandemeulebroucke E, Balzarini J, Schols D (2008) Safety concerns for the potential use of cyanovirin-N as a microbicidal anti-HIV agent. Int J Biochem Cell Biol 40: 2802–2814. doi: 10.1016/j.biocel.2008.05.023.
CrossRef • PubMed/NCBI • Google Scholar
38. Fichorova RN, Rheinwald JG, Anderson DJ (1997) Generation of papillomavirus-immortalized cell lines from normal human ectocervical, endocervical, and vaginal epithelium that maintain expression of tissue-specific differentiation proteins. Biol Reprod 57: 847–855. doi: 10.1095/biolreprod57.4.847.
CrossRef • PubMed/NCBI • Google Scholar
39. Fichorova RN, Anderson DJ (1999) Differential expression of immunobiological mediators by immortalized human cervical and vaginal epithelial cells. Biol Reprod 60: 508–514. doi: 10.1095/biolreprod60.2.508.
CrossRef • PubMed/NCBI • Google Scholar
40. Yamamoto HS, Xu Q, Fichorova RN (2013) Homeostatic properties of Lactobacillus jensenii engineered as a live vaginal anti-HIV microbicide. BMC Microbiol 13: 4. doi: 10.1186/1471-2180-13-4.
CrossRef • PubMed/NCBI • Google Scholar
41. Perrier S, Darakhshan F, Hajduch E (2006) IL-1 receptor antagonist in metabolic diseases: Dr Jekyll or Mr Hyde? FEBS Lett 580: 6289–6294. doi: 10.1016/j.febslet.2006.10.061.
CrossRef • PubMed/NCBI • Google Scholar
42. Poli G, Kinter AL, Fauci AS (1994) Interleukin 1 induces expression of the human immunodeficiency virus alone and in synergy with interleukin 6 in chronically infected U1 cells: inhibition of inductive effects by the interleukin 1 receptor antagonist. Proc Natl Acad Sci U S A 91: 108–112. doi: 10.1073/pnas.91.1.108.
CrossRef • PubMed/NCBI • Google Scholar
43. Ghadimi D, de Vrese M, Heller KJ, Schrezenmeir J (2010) Lactic acid bacteria enhance autophagic ability of mononuclear phagocytes by increasing Th1 autophagy-promoting cytokine (IFN-gamma) and nitric oxide (NO) levels and reducing Th2 autophagy-restraining cytokines (IL-4 and IL-13) in response to

Mycobacterium tuberculosis antigen. Int Immunopharmacol 10: 694–706. doi: 10.1016/j.intimp.2010.03.014.
CrossRef • PubMed/NCBI • Google Scholar

44. Liu Y, Fatheree NY, Mangalat N, Rhoads JM (2010) Human-derived probiotic Lactobacillus reuteri strains differentially reduce intestinal inflammation. Am J Physiol Gastrointest Liver Physiol 299: G1087–1096. doi: 10.1152/ajpgi.00124.2010.
CrossRef • PubMed/NCBI • Google Scholar
45. Snel J, Vissers YM, Smit BA, Jongen JM, van der Meulen ET, et al. (2011) Strain-specific immunomodulatory effects of Lactobacillus plantarum strains on birch-pollen-allergic subjects out of season. Clin Exp Allergy 41: 232–242. doi: 10.1111/j.1365-2222.2010.03650.x.
CrossRef • PubMed/NCBI • Google Scholar
46. Vissers YM, Snel J, Zuurendonk PF, Kleerebezem M, Wichers HJ, et al. (2011) Lactobacillus strains differentially modulate cytokine production by hPBMC from pollen-allergic patients. FEMS Immunol Med Microbiol 61: 28–40. doi: 10.1111/j.1574-695x.2010.00741.x.
CrossRef • PubMed/NCBI • Google Scholar
47. Inamine A, Sakurai D, Horiguchi S, Yonekura S, Hanazawa T, et al. (2012) Sublingual administration of Lactobacillus paracasei KW3110 inhibits Th2-dependent allergic responses via upregulation of PD-L2 on dendritic cells. Clin Immunol 143: 170–179. doi: 10.1016/j.clim.2012.01.008.
CrossRef • PubMed/NCBI • Google Scholar
